# Supplementary material for: Design of a MAPK signalling cascade balances energetic cost versus accuracy of information transmission
Source: Nat Commun. 2020 Jul 13;11:3494. doi: 10.1038/s41467-020-17276-4 (PMC7359329; doi:10.1038/s41467-020-17276-4)
Supplement: Supplementary file 1 — Supplementary Information [file 41467_2020_17276_MOESM1_ESM.pdf]

Supplementary Information

Design of a MAPK signalling cascade balances energetic cost versus accuracy of information transmission

Anders *et al*.

This file includes:

Supplementary Notes 1-5
Supplementary Figures 1-15
Supplementary Table 1
Supplementary References

Supplementary Notes

Supplementary Note 1: Fisher and mutual information

Calculation of Fisher information: If θ is an unknown deterministic parameter which is to be estimated from the measurement of parameter x, distributed according to a probability density function f(x;θ), the variance of any unbiased estimator of θ is then bounded by the reciprocal of the Fisher information $F(\theta)$

$$\mathrm{var}(\theta) \geq \frac{1}{F(\theta)} \tag{1}$$

where $F(\theta)$=$\mathrm{E}\left[\left(\frac{\delta \mathrm{l}(\mathrm{x};\theta)}{\delta\theta}\right)^2\right]$; $l(\mathrm{x};\theta) = -\log\left(P(\mathrm{x};\theta)\right)$ and E denotes the expectation value. Hence, higher value of $I(\theta)$ would reduce the variance leading to a better precision in estimating the value of θ. In our case, θ is the pheromone concentration, on the log scale.

As the experimental means and variances at different pheromone concentrations are known, we can calculate Fisher information for every input. Here the mean and standard deviation (s.d.) are calculated from the response curve at each dose. We assume that the distribution at each dose is normal with mean μ and s.d. = σ in order to take derivative of the log-likelihood function. Fisher information is given by

$$F(\theta) = E\left[\left(\frac{\partial l(x;\theta)}{\partial\theta}\right)^2\right]; l(x;\theta) = -log[P(x;\theta)] \tag{2}$$

where x and θ represent the response and the dose (pheromone concentration) respectively. $P(x;\theta)$ is the probability distribution of the response x at a particular value of the dose θ, with mean $\mu(\theta)$ and s.d. $\sigma(\theta)$. We assume that the distribution at each dose is normal. We can numerically calculate the derivative with respect to the dose by taking small step θ of step size $\Delta\theta$ in the following way:

$$\frac{\Delta l}{\Delta\theta} = \frac{l(x;\theta+\Delta\theta) - l(x;\theta)}{\Delta\theta}; \Delta\theta = 10^{-5}; \tag{3}$$

$$P(x;\theta) = \frac{1}{\sqrt{2\pi\sigma(\theta)^2}} exp\left[\frac{-\left(x-\mu(\theta)\right)^2}{2\sigma(\theta)^2}\right]; \tag{4}$$

$$l(x;\theta) = -log[P(x;\theta)] = \frac{1}{2}log(2\pi\sigma(\theta)^2) + \frac{\left(x-\mu(\theta)\right)^2}{2\sigma(\theta)^2}; \tag{5}$$

$$l(x;\theta+\Delta\theta) = -log[P(x;\theta+\Delta\theta)] = \frac{1}{2}log(2\pi\sigma(\theta+\Delta\theta)^2) + \frac{\left(x-\mu(\theta+\Delta\theta)\right)^2}{2\sigma(\theta+\Delta\theta)^2}; \tag{6}$$

$$F(\theta) = \int_0^\infty \left(\frac{\Delta l}{\Delta\theta}\right)^2 P(x;\theta)dx = \int_0^\infty \left(\frac{l(x;\theta+\Delta\theta) - l(x;\theta)}{\Delta\theta}\right)^2 P(x;\theta)dx \tag{7}$$

$$\Rightarrow F(\theta) = \frac{1}{\Delta\theta^2}\int_0^\infty \left(\frac{1}{2}log(2\pi\sigma(\theta+\Delta\theta)^2) + \frac{\left(x-\mu(\theta+\Delta\theta)\right)^2}{2\sigma(\theta+\Delta\theta)^2} - \frac{1}{2}log(2\pi\sigma(\theta)^2) - \frac{\left(x-\mu(\theta)\right)^2}{2\sigma(\theta)^2}\right)^2 P(x;\theta)dx \tag{8}$$

First, $\mu(\theta)$ and $\sigma(\theta)$ of the output at experimentally measured discrete values of dose θ are locally fitted (with a window of 3 pheromone concentrations in log scale) by cubic spline with $10^5(\Delta\theta = 10^{-5})$ points, and the Fisher information at a particular θ is then calculated as shown in the above formula.

Functional relationship of Fisher information with amplification and noise: The probability distributions are assumed to be normal at each value of the input

$$p(\mathrm{x} \mid \theta) = \frac{1}{\sqrt{2\pi\sigma(\theta)^2}} e^{-\frac{1}{2}\left(\frac{x-\mu(\theta)}{\sigma(\theta)}\right)^2} \tag{9}$$

$$\frac{-\partial}{\partial\theta} log[p(\mathrm{x} \mid \theta)] = \frac{(x-\mu)}{\sigma^2}\frac{\partial\mu}{\partial\theta} + \frac{1}{\sigma}\left(\frac{(x-\mu)^2}{\sigma^2} - 1\right)\frac{\partial\sigma}{\partial\theta} \tag{10}$$

$$\left(\frac{\partial}{\partial\theta} log[p(\mathrm{x} \mid \theta)]\right)^2 = \frac{(x-\mu)^2}{\sigma^4}\left(\frac{\partial\mu}{\partial\theta}\right)^2 + \frac{1}{\sigma^2}\left(\frac{(x-\mu)^4}{\sigma^4} + 1 - 2\frac{(x-\mu)^2}{\sigma^2}\right)\left(\frac{\partial\sigma}{\partial\theta}\right)^2 + \frac{1}{\sigma^3}\left(\frac{(x-\mu)^3}{\sigma^2} - (x-\mu)\right)\left(\frac{\partial\mu}{\partial\theta}\right)\left(\frac{\partial\sigma}{\partial\theta}\right) \tag{11}$$

Taking average over *x* while the cross term vanishes due to zero moments of the odd exponents for normal distribution and $\langle(x-\mu)^4\rangle = 3\sigma^4; \langle(x-\mu)^2\rangle = \sigma^2$, we obtain

$$\left\langle\left(\frac{\partial}{\partial\theta} log[p(\mathrm{x} \mid \theta)]\right)^2\right\rangle = \frac{1}{\sigma^2}\left[\left(\frac{\partial\mu}{\partial\theta}\right)^2 + 2\left(\frac{\partial\sigma}{\partial\theta}\right)^2\right] = \frac{1}{\eta^2}\left[\frac{1}{\mu^2}\left(\frac{\partial\mu}{\partial\theta}\right)^2 + \frac{2}{\mu^2}\left(\frac{\partial\sigma}{\partial\theta}\right)^2\right] \tag{12}$$

We further assume that noise η decreases with the mean proportional to square root of μ:

$$\sigma^2 \sim c\mu \rightarrow \left(\frac{\partial\sigma}{\partial\theta}\right)^2 \sim \frac{c^2}{4c\mu}\left(\frac{\partial\mu}{\partial\theta}\right)^2 \tag{13}$$

Hence, the Fisher information is given by

$$F(\theta) = \left\langle\left(\frac{\partial}{\partial\theta} log[p(\mathrm{x} \mid \theta)]\right)^2\right\rangle \approx \frac{1}{\eta^2}\left(1 + \frac{c}{2\mu}\right)\left(\frac{\partial log\mu}{\partial\theta}\right)^2 \tag{14}$$

Calculation of mutual information: Generally, mutual information can be quantified as

$$I(\mathrm{R;S}) = \iint_{\mathrm{SR}} P\,(\mathrm{R} \mid \mathrm{S})P(S)\log_2\frac{P(\mathrm{R} \mid \mathrm{S})}{P(R)}\,\mathrm{dRdS} \tag{15}$$

P(*R*|S) corresponds to the conditional probability of a particular response *R* for a signal *S*. Thus, *P(R|S)* can be determined from single-cell measurements of the reporter output for an isogenic cell population at different signals. *P(R)* indicates the probability distribution for all values of S. The signal distribution, *P(S)*, reflects the frequencies of the specific signal values experienced by the population. Since here we do not have any prior information about the input signal distribution, we assumed that all signals are equally probable (that is, *P(S)* is uniform). Note that in general mutual information and Fisher information are connected, as higher Fisher information at a particular input

also means higher local mutual information about the input

$$I(\theta;x) \geq H(\theta) - \int d\,\theta'\rho(\theta')\frac{1}{2}\log\left(\frac{2\pi e}{I(\theta')}\right) \tag{16}$$

where θ is equivalent to S (pheromone concentration) and R is equivalent to the response x. $\rho(\theta)$ describes the input distribution, which is assumed here to be uniform, and $H(\theta)$ denotes the entropy of the input.

While estimating Fisher information we assume the distributions to be normal, we can also calculate local mutual information where we do not need to make any assumptions about the distributions. Mutual information is given by

$$I(x;\theta) = \iint_{x\theta} P\,(x \mid \theta)P(x)log_2\frac{P(x \mid \theta)}{P(x)}dxd\theta \tag{17}$$

$$I(x;\theta) = \int_x P\,(x)P(x)log_2P(x)dx - \iint_{x\theta} P\,(\theta)P(x \mid \theta)log_2P(x \mid \theta)dxd\theta \tag{18}$$

$$I(x;\theta) = E(x) - E(x \mid \theta) \tag{19}$$

The total entropy $E(x)$ is calculated by binning the unconditional distribution $P(x)$ into 20 bins from the single cell measurements by fluorescence microscopy. Similarly, the conditional entropy $E(x \mid \theta)$ is also calculated from the conditional distribution $p(x \mid \theta)$ where the distribution is quantified at different value of the pheromone dose θ The mutual information is the reduction in the conditional entropy compared to the total entropy. The input distribution $P(\theta)$ is assumed to be uniform while calculating the mutual information. The basic results remain the same irrespective of the number of bins or different sample size [1] (not shown). The local mutual information is calculated by taking a sliding window having three pheromone concentrations. The same procedure is followed to calculate Fisher and mutual information from the simulated data.

Supplementary Note 2: Models of the pheromone response pathway

Simple two-stage model with negative feedback for analytic noise calculation: In the schematic diagram for a two stage phosphorylation cascade shown below, S corresponds to the signalling molecule. $X_1(X_2)$ represents the dephosphorylated (phosphorylated) form of the upstream signalling protein X; $Y_1(Y_2)$ represents the dephosphorylated (phosphorylated) form of the downstream signalling protein Y.

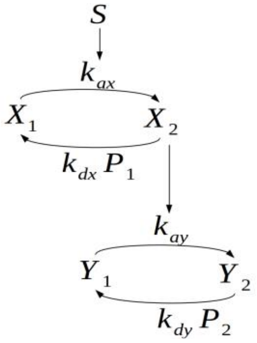

With feedback present at the upper level, the dynamics of the system can be written as

$$\frac{dx_2}{dt} = k_{ax}X_TS - \{k_{ax}S + k_{dx}g(y_2)P_1\}x_2 \tag{20}$$

$$\frac{dy_2}{dt} = k_{ay}Y_Tx_2 - \{k_{ay}x_2 + k_{dy}P_2\}y_2 \tag{21}$$

Here, x_2 and y_2 correspond to the concentrations of activated receptor Ste2 and activated Fus3, respectively, and $g(y)$ indicates transcriptional activation of a negative regulator (Sst2) with maximum induction P_1. We assume that enzyme concentrations are below saturation so that we can linearize the equations. Expanding the equations to the first order around steady state of active X and active Y from the equation above described by x_{2s}, y_{2s} respectively and adding Gaussian white noise terms yields

$$\frac{d\Delta x_2}{dt} = -\{k_{ax}S + k_{dx}g(y_{2s})P_1\}\Delta x_2 - (k_{dx}G(y_{2s})P_1\}\Delta y_2 + \xi_1(t) \tag{22}$$

$$\frac{d\Delta y_2}{dt} = k_{ay}(Y_T - y_{2s})\Delta x_2 - \{k_{ay}x_{2s} + k_{dy}P_2\}\Delta y_2 + \xi_2(t) \tag{23}$$

Induction of the negative regulator

$$g(y_2) = \frac{(y_2/k_l)^2}{1 + (y_2/k_l)^2}, G(y_{2s}) = \left(\frac{\partial g}{\partial y_2}\right)_{y_{2s}} \tag{24}$$

and

$$\langle \xi_1(t_1)\rangle = 0, \langle \xi_2(t_1)\rangle = 0 \tag{25}$$

$$\langle \xi_1(t_1)\xi_1(t_2)\rangle = q_x\delta(t_1 - t_2), \langle \xi_2(t_1)\xi_2(t_2)\rangle = q_y\delta(t_1 - t_2) \tag{26}$$

The covariance matrix is calculated by solving the matrix equation

$$A \cdot \sigma + \sigma \cdot A^T - B = 0 \tag{27}$$

where

$$A = \begin{bmatrix} -\{k_{ax}S + k_{dx}g(y_{2s})P_1\} & -\{k_{dx}G(y_{2s})P_1\} \\ k_{ay}(Y_T - y_{2s}) & -\{k_{ay}x_{2s} + k_{dy}P_2\} \end{bmatrix} \tag{28}$$

and

$$B = \begin{bmatrix} q_x & 0 \\ 0 & q_y \end{bmatrix} \tag{29}$$

The variances are given by

$$\sigma^2(x_2) = q_x \frac{d^2}{2(a+d)(ad-bc)} + q_x \frac{1}{2(a+d)} + q_y \frac{b^2}{2(a+d)(ad-bc)} \tag{30}$$

$$\sigma^2(y_2) = q_x \frac{c^2}{2(a+d)(ad-bc)} + q_y \frac{2}{2(a+d)} + q_y \frac{a^2}{2(a+d)(ad-bc)} \tag{31}$$

where

$$\mathrm{a}=\{k_{\mathrm{ax}}\mathrm{S}+\mathrm{k}_{\mathrm{dx}}g(y_{2s})P_1\},\mathrm{b}=-\{k_{\mathrm{dx}}G(y_{2s})P_1\} \tag{32}$$

$$\mathrm{c}=\mathrm{k}_{\mathrm{ay}}(Y_T - y_{2s}),\mathrm{d}=\{k_{\mathrm{ay}}x_{2s}+\mathrm{k}_{\mathrm{dy}}P_2\} \tag{33}$$

$$q_x = 2ax_{2s}; q_y = 2dy_{2s}; \tag{34}$$

and strength for the negative feedback is given by

$$b = k_{dx}G(y_{2s})P_1 = k_{dx}\frac{2y_{2s}k_l^2}{(y_{2s}^2 + k_l^2)^2}P_1 \tag{35}$$

Substituting in the equations for variances we get an expression

$$\sigma^2(y_2) = y_{2s}\frac{a + \frac{bc}{a+d}}{a + \frac{bc}{a}} + \frac{ak_{ay}^2(Y_T - y_{2s})^2}{(a+d)(ad+bc)}x_{2s} = y_{2s}\left[\frac{a + \frac{bc}{a+d}}{a + \frac{bc}{a}} + \frac{ak_{dy}^2P_2^2}{(a+d)(ad+bc)}\left(\frac{y_{2s}}{x_{2s}}\right)\right] \tag{36}$$

The first term represents the intrinsic noise in y and the second term reflects the noise propagating from the upstream stage. Both noise terms would be reduced in the presence of feedback as b is nonzero.

If the feedback is present at lower level, i.e. when the phosphatase Msg5 is induced

where $g(y)$ here corresponds to the induction of the negative regulator Msg5. The linearized equation around steady state with the addition of Gaussian white noise

$$\frac{dx_2}{dt} = k_{ax}X_TS - \{k_{ax}S + k_{dx}P_1\}x_2 \tag{37}$$

$$\frac{dy_2}{dt} = k_{ay}Y_Tx_2 - \{k_{ay}x_2 + k_{dy}g(y_2)P_2\}y_2 \tag{38}$$

$$\frac{d\Delta x_2}{dt} = -\{k_{ax}S + k_{dx}g(y_{2s})P_1\}\Delta x_2 - \xi_1(t) \quad (39)$$

$$\frac{d\Delta y_2}{dt} = k_{ay}(Y_T - y_{2s})\Delta x_2 - \{k_{ay}x_{2s} + k_{dy}P_2 + k_{dy}G(y_{2s})P_2\}\Delta y_2 + \xi_2(t) \quad (40)$$

By solving for the variance with the similar method as before, we get an expression for the variance

$$\sigma^2(y_2) = y_{2s}\left[\frac{d}{d+b} + \frac{k_{dy}^2 P_2^2 g^2(y_{2s})}{(d+b)(a+d+b)}\left(\frac{y_{2s}}{x_{2s}}\right)\right]; \begin{array}{c} a=\{k_{ax}S+k_{dx}P_1\}, b=\{k_{dy}G(y_{2s})P_2\} \\ c=k_{ay}(Y_T - y_{2s}), d=\{k_{ay}x_{2s}+k_{dy}g(y_{2s})P_2\} \\ q_x = 2ax_{2s}; q_y = 2dy_{2s}; \end{array} \quad (41)$$

In the presence of feedback *b*, the noise will be lower at a particular steady state compared to the case when *b* is zero. So, feedback at the lower level would be able to reduce both the intrinsic noise and the noise propagated from an upper level.

<u>A detailed model used for simulating the pathway output and calculating the information transmission capacity:</u> The pheromone response pathway is modelled as a two-stage phosphorylation cascade. The MAPK Fus3 is phosphorylated following activation of the receptor Ste2. Doubly phosphorylated Fus3 in turn activates the transcription factor Ste12 by phosphorylation. Activated Ste12 induces transcription of *FUS1* which is considered as the reporter output of the pathway. Furthermore, activated Ste12 transcriptionally activates Fus3 and Ste2, constituting two positive feedback loops, and the phosphatase Msg5 as well as the RGS Sst2, constituting negative feedback loops. Differential equations are constructed based on mass action kinetics. Transcriptional induction is assumed to follow Michaelis-Menten kinetics. De-activation of the receptor relies on the GTP hydrolysis whereas phosphorylation reactions consume ATP, resulting in the energy cost in signal transduction. In order to characterize noise, the production rates and the initial protein concentrations are selected randomly around the central values from log-normal distributions with standard deviation (s.d.) of 30%. Noise propagated through the pathway is quantified as coefficient of variation (CV) of the final *FUS1* transcriptional output or active Fus3 ($FUS3_{PP}$). The accuracy of the signal transduction is measured by calculating Fisher and mutual information from the distribution of the outputs. The ODEs are solved using CVode [2] interfaced with MATLAB.

The set of reactions is given by:

Activation of the receptor by pheromone

$$\overset{x_1}{S_\alpha} + \overset{x_2}{STE2} \underset{K_{-S}}{\overset{k_S}{\rightleftharpoons}} \overset{x_3}{STE2_a}; K_{-S} = k_{-S}SST2 \quad (42)$$

Phosphorylation of Fus3

$$\overset{x_4}{FUS3} + \overset{x_3}{STE2_a} \underset{k_{-p}}{\overset{k_p}{\rightleftharpoons}} \overset{x_5}{FUS3-STE2_a} \overset{\beta_p}{\longrightarrow} \overset{x_6}{FUS3_p} + \overset{x_3}{STE2_a} \tag{43}$$

$$\overset{x_6}{FUS3_p} + \overset{x_3}{STE2_a} \underset{k_{-p}}{\overset{k_p}{\rightleftharpoons}} \overset{x_7}{FUS3_p-STE2_a} \overset{\beta_p}{\longrightarrow} \overset{x_8}{FUS3_{pp}} + \overset{x_3}{STE2_a} \tag{44}$$

De-phosphorylation of Fus3 by Msg5

$$\overset{x_6}{FUS3_p} + \overset{x_9}{MSG5} \underset{k_{-u}}{\overset{k_u}{\rightleftharpoons}} \overset{x_{10}}{FUS3_p-MSG5} \overset{\beta_u}{\longrightarrow} \overset{x_4}{FUS3} + \overset{x_9}{MSG5} \tag{45}$$

$$\overset{x_8}{FUS3_{pp}} + \overset{x_9}{MSG5} \underset{k_{-u}}{\overset{k_u}{\rightleftharpoons}} \overset{x_{11}}{FUS3_{pp}-MSG5} \overset{\beta_p}{\longrightarrow} \overset{x_6}{FUS3_p} + \overset{x_9}{MSG5} \tag{46}$$

De-phosphorylation of Fus3 by Ptp3

$$\overset{x_8}{FUS3_{pp}} + \overset{x_{12}}{PTP3} \underset{k_{-u}}{\overset{k_u}{\rightleftharpoons}} \overset{x_{13}}{FUS3_{pp}-PTP3} \overset{\beta_u}{\longrightarrow} \overset{x_6}{FUS3_p} + \overset{x_{12}}{PTP3} \tag{47}$$

De-phosphorylation of Fus3 by Ptp2

$$\overset{x_8}{FUS3_{pp}} + \overset{x_{14}}{PTP2} \underset{k_{-u}}{\overset{k_u}{\rightleftharpoons}} \overset{x_{15}}{FUS3_{pp}-PTP2} \overset{\beta_u}{\longrightarrow} \overset{x_6}{FUS3_p} + \overset{x_{14}}{PTP2} \tag{48}$$

Activation of Ste12 through phosphorylation by Fus3-PP

$$\overset{x_{16}}{STE12} + \overset{x_8}{FUS3_{pp}} \underset{k_{-p}}{\overset{k_p}{\rightleftharpoons}} \overset{x_{17}}{FUS3_{pp}-STE12} \overset{\beta_p}{\longrightarrow} \overset{x_{18}}{STE12_p} + \overset{x_8}{FUS3_{pp}} \tag{49}$$

De-phosphorylation of Ste12

$$\overset{x_{18}}{STE12_p} + \overset{x_{19}}{Phos} \underset{k_{-u}}{\overset{k_u}{\rightleftharpoons}} \overset{x_{20}}{STE12_p-Phos} \overset{\beta_u}{\longrightarrow} \overset{x_{16}}{STE12} + \overset{x_{19}}{Phos} \tag{50}$$

Transcriptional activation of FUS1 by Ste12-P

$$G \overset{\beta_{fus1}}{\longrightarrow} \overset{x_{21}}{FUS1} \overset{\gamma_{fus1}}{\longrightarrow} \phi, \beta_{fus1} = \beta_0^{fus1} + \beta_{induced}^{fus1} \frac{STE12_p^2}{K_{fus1}^2 + STE12_p^2} \tag{51}$$

Transcriptional activation of Fus3 by Ste12-P

$$G \overset{\beta_{fus3}}{\longrightarrow} \overset{x_4}{FUS3} \overset{\gamma_{fus3}}{\longrightarrow} \phi, \beta_{fus3} = \beta_0^{fus3} + \beta_{induced}^{fus3} \frac{STE12_p^2}{K_{fus3}^2 + STE12_p^2} \tag{52}$$

Transcriptional activation of Ste2 by Ste12-P

$$G \overset{\beta_{ste2}}{\longrightarrow} STE\,2 \overset{x_2\ \gamma_{ste2}}{\longrightarrow} \phi, \beta_{ste2} = \beta_0^{ste2} + \beta_{duced}^{ste2} \frac{STE12_p^2}{K_{ste2}^2 + STE12_p^2} \quad (53)$$

Transcriptional activation of Sst2 by Ste12-P

$$G \overset{\beta_{sst2}}{\longrightarrow} SST\,2 \overset{x_{22}\ \gamma_{ste2}}{\longrightarrow} \phi, \beta_{sst2} = \beta_0^{sst2} + \beta_{induced}^{sst2} \frac{STE12_p^2}{K_{sst2}^2 + STE12_p^2} \quad (54)$$

Transcriptional activation of Msg5 by Ste12-P

$$G \overset{\beta_{msg5}}{\longrightarrow} MSG\,5 \overset{x_9\ \gamma_{MSG5}}{\longrightarrow} \phi, \beta_{msg5} = \beta_0^{msg5} + \beta_{induced}^{msg5} \frac{STE12_p^2}{K_{msg5}^2 + STE12_p^2} \quad (55)$$

Production of Ptp3

$$G \overset{\beta_{ptp3}}{\longrightarrow} PTP\,3 \overset{x_{12}\ \gamma_{ptp3}}{\longrightarrow} \phi, \beta_{ptp3} = \beta_0^{ptp3} \quad (56)$$

The corresponding dynamic equations are as follows

$$\frac{dx_1}{dt} = -k_s x_1 x_2 + k_{-s} x_3 \quad (57)$$

$$\frac{dx_2}{dt} = -k_s x_1 x_2 + k_{-s} x_3 + \beta_{ste2} - \gamma_{ste2} x_2 \quad (58)$$

$$\frac{dx_3}{dt} = k_s x_1 x_2 - k_{-s} x_3 - k_p x_4 x_3 + k_{-p} x_5 + \beta_p x_5 - k_p x_6 x_3 + k_{-p} x_7 + \beta_p x_7 - \gamma_{ste2} x_3 \quad (59)$$

$$\frac{dx_4}{dt} = -k_p x_4 x_3 + k_{-p} x_5 + \beta_u x_{10} + \beta_{fus3} - \gamma_{fus3} x_4 \quad (60)$$

$$\frac{dx_5}{dt} = k_p x_4 x_3 - k_{-p} x_5 - \beta_u x_5 - \gamma_{fus3} x_5 \quad (61)$$

$$\frac{dx_6}{dt} = -k_p x_6 x_3 + k_{-p} x_7 + \beta_p x_5 + \beta_u x_{10} - k_u x_6 x_9 + k_{-u} x_{10} + \beta_u x_{11} + \beta_u x_{13} + \beta_u x_{15} - \gamma_{fus3} x_6 \quad (62)$$

$$\frac{dx_7}{dt} = k_p x_6 x_3 - k_{-p} x_7 - \beta_p x_7 - \gamma_{fus3} x_7 \quad (63)$$

$$\frac{dx_8}{dt} = -k_u x_8 x_9 + k_{-u} x_{11} + \beta_p x_7 - k_u x_8 x_{12} + k_{-u} x_{13} - k_u x_8 x_{14} + k_{-u} x_{15} - k_p x_8 x_{16} + k_{-p} x_{17} + \beta_p x_{17} \quad (64)$$

$$\frac{dx_9}{dt} = -k_u x_6 x_9 + k_{-u} x_{10} + \beta_u x_{10} - k_u x_8 x_9 + k_{-u} x_{11} + \beta_u x_{11} + \beta_{msg5} - \gamma_{msg5} x_9 \quad (65)$$

$$\frac{dx_{10}}{dt} = k_u x_6 x_9 - k_{-u} x_{10} - \beta_u x_{10} - \gamma_{fus3} x_{10} \quad (66)$$

$$\frac{dx_{11}}{dt} = k_u x_8 x_9 - k_{-u} x_{11} - \beta_u x_{11} - \gamma_{fus3} x_{11} \quad (67)$$

$$\frac{dx_{12}}{dt} = -k_u x_8 x_{12} + k_{-u} x_{13} + \beta_u x_{13} + \beta_{ptp3} - \gamma_{ptp3} x_{12} \quad (68)$$

$$\frac{dx_{13}}{dt} = k_u x_8 x_{12} - k_{-u} x_{13} - \beta_u x_{13} - \gamma_{fus3} x_{13} \quad (69)$$

$$\frac{dx_{14}}{dt} = -k_u x_8 x_{14} + k_{-u} x_{15} + \beta_u x_{15} + \beta_{ptp2} - \gamma_{ptp2} x_{14} \quad (70)$$

$$\frac{dx_{15}}{dt} = k_u x_8 x_{14} - k_{-u} x_{15} - \beta_u x_{15} - \gamma_{fus3} x_{15} \quad (71)$$

$$\frac{dx_{16}}{dt} = -k_p x_8 x_{16} + k_{-p} x_{17} + \beta_u x_{20} \quad (72)$$

$$\frac{dx_{17}}{dt} = k_p x_8 x_{16} - k_{-p} x_{17} - \beta_p x_{17} \quad (73)$$

$$\frac{dx_{18}}{dt} = -k_u x_{18} x_{19} + k_{-p} x_{20} + \beta_p x_{17} \quad (74)$$

$$\frac{dx_{19}}{dt} = -k_u x_{18} x_{19} + k_{-u} x_{20} + \beta_u x_{20} \quad (75)$$

$$\frac{dx_{20}}{dt} = k_u x_{18} x_{19} - k_{-u} x_{20} - \beta_u x_{20} \quad (76)$$

$$\frac{dx_{21}}{dt} = \beta_{fus1} - \gamma_{fus1} x_{21} \quad (77)$$

$$\frac{dx_{22}}{dt} = \beta_{sst2} - \gamma_{sst2} x_{22} \quad (78)$$

Values of the rate constants:

$$k_s = 0.03nM^{-1}s^{-1}; k_{-s} = 0.003s^{-1};$$
$$k_p = 0.01nM^{-1}s^{-1}; k_{-p} = 10s^{-1}; \beta_p = 30s^{-1};$$
$$k_u = 0.004nM^{-1}s^{-1}; k_{-u} = 2.5s^{-1}; \beta_u = 30s^{-1};$$
$$\beta_0^{fus1} = 0.01nMs^{-1}; \beta_{induced}^{fus1} = 10nMs^{-1}; K_{fus1} = 100nM; \gamma_{fus1} = 0.0001s^{-1};$$
$$\beta_0^{fus3} = 0.2nMs^{-1}; \beta_{induced}^{fus3} = 2.5nMs^{-1}; K_{fus3} = 100nM; \gamma_{fus3} = 0.0005s^{-1};$$
$$\beta_0^{ste2} = 2nMs^{-1}; \beta_{induced}^{ste2} = 50nMs^{-1}; K_{ste2} = 10nM; \gamma_{ste2} = 0.0005s^{-1};$$
$$\beta_0^{msg5} = 0.02nMs^{-1}; \beta_{induced}^{msg5} = 4.5nMs^{-1}; K_{msg5} = 700nM; \gamma_{msg5} = 0.0005s^{-1};$$
$$\beta_0^{sst2} = 0.06nMs^{-1}; \beta_{induced}^{sst2} = 6nMs^{-1}; K_{sst2} = 8nM; \gamma_{sst2} = 0.0005s^{-1};$$
$$\beta_0^{ptp3} = 0.2nMs^{-1}; \gamma_{ptp3} = 0.0005s^{-1};$$
$$\beta_0^{ptp2} = 0.08nMs^{-1}; \gamma_{ptp2} = 0.0005s^{-1}; Ste12_{TOT} = 2000nM; Phos_{ste12} = 100nM$$

Protein concentrations: *FUS3* = 200 nM; *STE2* = 4000 nM; *STE12* = 1200 nM; *SST2* = 120 nM; *MSG5* = 40 nM.

These values were initially chosen based on Refs[3–5] and subsequently adjusted to reproduce dose response and noise characteristics of the WT, *msg5Δ* and *sst2Δ* responses and the relative induction sensitivities of the feedback regulators as measured by RNA sequencing experiments.

For this model, the total ATP+GTP consumption rate in signal transduction is given by

$$Energy/min = k_{-s}[SST2][STE2_a] + \beta_p[FUS3_p - STE2_a] + \beta_p[FUS3 - STE2_a] \quad (79)$$

Stochastic simulations of the model at varying pheromone concentration were performed for a population of 500 cells. Cell-to-cell variability was added to each production rate, with 60% extrinsic and 40% intrinsic noise.

Different induction sensitivities of negative feedback regulators Msg5 and Sst2 were attained by varying the respective K_D values (K_{msg5}, K_{sst2}) for binding of Ste12-P to their promoters. Note that modified K_D values not only resulted in changed EC50s for the respective gene/protein, but also in changed (saturating) levels of protein production.

Supplementary Note 3: Comparison of energy consumptions for negative feedback induction at different stages of the cascade

A two-stage signalling cascade can be simplified as

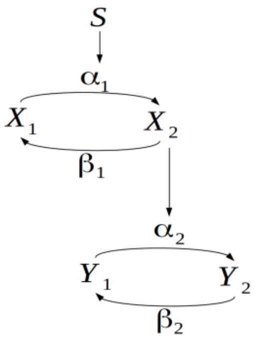

where active Y (Y_2) is the signalling output of the cascade and active X (X_2) is the activity at the upper stage. Then the energy consumption rate at steady state is given by

$$E = \beta_1 x_{2s} + \beta_2 y_{2s} \tag{80}$$

The steady states of active X and active Y are

$$x_{2s} = \frac{\alpha_1 s}{\alpha_1 s + \beta_1} X_{TOT}; y_{2s} = \frac{\alpha_2 x_{2s}}{\alpha_2 x_{2s} + \beta_2} Y_{TOT} \tag{81}$$

If now the dissipation at lower stage is increased *n* times, i.e. $\beta_2' = n\beta_2$,

the new steady state of active Y is

$$y'_{2s} = \frac{\alpha_2 x_{2s}}{\alpha_2 x_{2s} + n\beta_2} Y_{TOT} \tag{82}$$

In order to maintain the steady-state signalling output at the same level, new steady-state value of activity at the upper stage should become $x_{2s}' = n x_{2s}$

This new steady state of X_2 in our model can be attained by increasing the input *S*, resulting in increased energy consumption

$$E' = \beta_1 x_{2s}' + \beta_2' y_{2s} = \beta_1 (n x_{2s}) + (n\beta_{2_1}) y_{2s} = nE; \tag{83}$$

$$\Rightarrow \frac{E'}{E} = n; \tag{84}$$

In contrast, if dissipation at the upper stage is increased by *n* times, i.e. $k_{d_1}' = n k_{d_1}$, the new steady state of active X is

$$x'_{2s} = \frac{\alpha_1 s}{\alpha_1 s + n\beta_1} X_{TOT} \tag{85}$$

In order to maintain the same steady state, we can adjust $s \rightarrow ns$ without entailing any extra energy consumption at lower stage, hence the new energy consumption

$$E' = \beta_1' x_{2s} + \beta_2 y_{2s} = (n\beta_1) x_{2s} + \beta_2 y_{2s}; \tag{86}$$

$$\Rightarrow \frac{E'}{E} < n; \tag{87}$$

The derivation implies that for such cascade the induction of the negative feedback at lower stage would lead to higher energy consumption compared to the feedback at upper stage.

Supplementary Note 4: Relation between Fisher information and energy consumption rate

<u>Relation for a simple one-stage phosphorylation cycle:</u> In the schematic diagram of a one-stage phosphorylation cycle shown below, S corresponds to the signalling molecule. $X_1 (X_2)$ represents the dephosphorylated (phosphorylated) form of the signalling protein X.

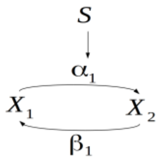

The dynamics of the active protein concentration is given by

$$\frac{dx_2}{dt} = \alpha_1 s X_T - \{\alpha_1 s + \beta_1\} x_2 \tag{88}$$

The steady state value of active X (x_2) is given by

$$x_{2s} = \frac{\alpha_1 s X_T}{\alpha_1 s + \beta_1} \tag{89}$$

The linearized equation around a steady state with adding a Gaussian white noise term

$$\frac{d\Delta x_2}{dt} = -\{\alpha_1 s + \beta_1\} \Delta x_2 + \xi_1(t) \tag{90}$$

$$\langle \xi_1(t_1) \rangle = 0 \tag{91}$$

$$\langle \xi_1(t_1) \xi_1(t_2) \rangle = 2\alpha_1 s X_T \delta(t_1 - t_2) \tag{92}$$

The variance in the active protein concentration

$$\sigma_x^2 = \langle \Delta x_2^2 \rangle = \frac{2\alpha_1 s X_T}{2(\alpha_1 s + \beta_1)} = x_{2s}; \Rightarrow \eta^2 = \frac{{\sigma_x}^2}{x_{2s}^2} = \frac{1}{x_{2s}} \tag{93}$$

The Fisher information can be written as in Text S3

$$F(s) = \left\langle \left(\frac{\partial}{\partial s} log[p(\mathrm{x} \mid \mathrm{s})] \right)^2 \right\rangle \approx \frac{1}{\eta^2} \left(1 + \frac{1}{2x_{2s}} \right) \left(\frac{\partial log(x_{2s})}{\partial s} \right)^2 \tag{94}$$

Substituting the above values of mean and variance yields

$$F(s) = \left(X_T \frac{s}{s + \beta_1/\alpha_1} + \frac{1}{2} \right) \left(\frac{\beta_1/\alpha_1}{s(s + \beta_1/\alpha_1)} \right)^2 \tag{95}$$

The energy consumption rate at steady state can be written as

$$E = \beta_1 x_{2s} \Rightarrow \beta_1 = \frac{\alpha_1 E s}{E_m - E}; E_m = \alpha_1 X_T s \tag{96}$$

Here, E_m is the maximum energy consumption rate possible at a particular value of stimulus (s), i.e., when dissipation rate is very high.

Converting the dissipation rate value by the energy consumption rate, we finally get a relationship between information and energy at a particular input value (s),

$$F = \frac{X_T}{s^2} (\overline{E})^2 \left(1 + \frac{1}{2X_T} - \overline{E} \right); \overline{E} = \frac{E}{E_m} \tag{97}$$

This shows that the information F increases with the energy consumption rate E. However, the information increases till $\overline{E} = \frac{2}{3}\left(1 + \frac{1}{2X_T}\right)$ where information attains a maximum value. If energy consumption due to dissipation is much higher than this value, information cannot be gained anymore by increasing energy consumption.

Similarly, information per unit energy is given by

$$\frac{F}{E} = \frac{1}{E_m} \frac{X_T}{s^2} (\overline{E}) \left(1 + \frac{1}{2X_T} - \overline{E} \right) \tag{98}$$

The information per energy reaches a maximum at $\overline{E} = \frac{1}{2}\left(1 + \frac{1}{2X_T}\right)$ which is lower than the energy for maximum information. From these expressions of information and information per energy, the integrated values can also be determined as

$$P = \int_{S_0}^{\infty} F(s) ds = \int_{S_0}^{\infty} \frac{X_T}{s^2} \left(\frac{a}{s + a} \right)^2 \left(1 + \frac{1}{2X_T} - \frac{s}{s + a} \right) ds \,; a = \frac{\beta_1}{\alpha_1} \tag{99}$$

Here, the dissipation rate is β_1 and without any loss of generality we can assume that dissipation rate is measured in the unit of α_1. Then, $\beta_1 = a$ in the units of α_1.

We assume that the number of total protein molecules X_T is large so that we can neglect the $\frac{1}{2X_T}$ term, yielding

$$P = \int_{s_0}^{\infty} X_T \frac{a^2}{s} \left(\frac{1}{s+a}\right)^3 ds \qquad (100)$$

here, s_0 signifies basal pathway activity.

The integrated information per energy is given by

$$R = \int_{s_0}^{\infty} \frac{F(s)}{E(s)} ds = \int_{s_0>0}^{\infty} \frac{1}{\alpha_1} \frac{a}{s^2} \left(\frac{1}{s+a}\right)^2 ds \qquad (101)$$

By integrating we finally obtain the expressions for total information (P) and total information per energy consumption (R) as a function of the dissipation rate a

$$P = \frac{X_T}{a}\left[log\left(1+\frac{a}{s_0}\right) - 1.5 + 2\frac{s_0}{a(a+s_0)} - \frac{s_0^2}{a(a+s_0)^2}\right] \qquad (102)$$

$$R = \frac{1}{\alpha_1 a^2}\left[-2log\left(1+\frac{a}{s_0}\right) + \frac{a}{s_0} + \frac{a}{a+s_0}\right] \qquad (103)$$

Supplementary figure 15A displays the normalized values of P and R as function of the dissipation rate for a basal level activity $s_0 = 0.4$. It shows that the information is indeed gained as the dissipation rate is increased. On the other hand, the information per energy consumption initially improves with higher dissipation rate and attains a maximum at around the value 0.5 indicating a trade-off between information gain and corresponding consumption of energy. However, the information starts to reduce slowly at high dissipation.

Relation for a simple two-stage phosphorylation cycle: In the schematic diagram of a two stage phosphorylation cascade shown below, S corresponds to the signalling molecule. $X_1(X_2)$ represents the dephosphorylated (phosphorylated) form of the upstream signalling protein X. $Y_1(Y_2)$ represents the dephosphorylated (phosphorylated) form of the downstream signalling protein Y.

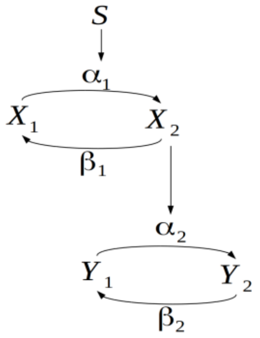

In this case, the dynamics of the active protein concentration is given by

$$\frac{dx_2}{dt} = \alpha_1 X_T s - \{\alpha_1 s + \beta_1\}x_2 \tag{104}$$

$$\frac{dy_2}{dt} = \alpha_2 Y_T x_2 - \{\alpha_2 x_2 + \beta_2\}y_2 \tag{105}$$

The steady state values of active X and active Y from the above equations are given by

$$x_{2s} = \frac{\alpha_1 s X_T}{(\alpha_1 s + \beta_1)}; y_{2s} = \frac{\alpha_2 x_{2s} Y_T}{(\alpha_2 x_{2s} + \beta_2)} \tag{106}$$

The linearized equation around the steady with adding Gaussian white noise terms

$$\frac{d\Delta x_2}{dt} = -\{\alpha_1 s + \beta_1\}\Delta x_2 + \xi_1(t) \tag{107}$$

$$\frac{d\Delta y_2}{dt} = \alpha_2(Y_T - y_{2s})\Delta x_2 - \{\alpha_2 x_{2s} + \beta_2\}\Delta y_2 + \xi_2(t) \tag{108}$$

$$\langle \xi_1(t_1) \rangle = 0 \tag{109}$$

$$\langle \xi_1(t_1)\xi_1(t_2) \rangle = 2\alpha_1 X_T s \delta(t_1 - t_2) \tag{110}$$

$$\langle \xi_2(t_1) \rangle = 0 \tag{111}$$

$$\langle \xi_2(t_1)\xi_2(t_2) \rangle = 2\alpha_2 Y_T x_{2s} \delta(t_1 - t_2) \tag{112}$$

The mean and variance in the active protein concentration

$$y_{2s} = Y_T \frac{X_T \frac{s}{s + a_1}}{a_2 + X_T \frac{s}{s + a_1}}; a_1 = \frac{\beta_1}{\alpha_1}; a_2 = \frac{\beta_2}{\alpha_2} \tag{113}$$

$$\sigma_y^2 = y_{2s}\left[1 + \frac{\beta_2^2}{\alpha_2(\alpha_1 s + \beta_1 + \alpha_2 x_{2s} + \beta_2)}\left(\frac{1}{Y_T}\right)\left(\frac{y_{2s}}{x_{2s}}\right)^2\right] \tag{114}$$

If Y_T is very high, the second term will be much small compared to one which would imply that $\eta_y^2 \sim \frac{1}{y_{2s}}$.

Hence, the Fisher information can be written as

$$F(s) = \left\langle \left(\frac{\partial}{\partial s} log[p(\mathrm{x} \mid \mathrm{s})]\right)^2 \right\rangle \approx \frac{1}{\eta_y^2}\left(1 + \frac{1}{2y_{2s}}\right)\left(\frac{\partial log(y_{2s})}{\partial s}\right)^2 \tag{115}$$

Substituting the above values of mean and variance yields

$$F(s) = \frac{Y_T}{a_2 a_2}\left(\frac{a_1 a_2}{a_2 + X_T}\right)^3 \frac{1}{s\left(s + \frac{a_1 a_2}{(a_2 + X_T)}\right)^3} \tag{116}$$

$$F(s) \approx \frac{Y_T}{a_2 a_2}\left(\frac{a_1 a_2}{X_T}\right)^3 \frac{1}{s\left(s + \frac{a_1 a_2}{X_T}\right)^3}; X_T \gg a_2 \tag{117}$$

Here, as mentioned before, the dissipation rate is $\beta_1(\beta_2)$ and we can assume that dissipation rate is

measured in the unit of $\alpha_1(\alpha_2)$.

Then, $\beta_1 = a_1$and $\beta_2 = a_2$

This shows that F is symmetric with respect to swapping of the values of the dissipation rates a_1 and a_2, if the number of molecule X_T is high. Since, the information only depends on the product of the dissipation rates, the value of the product will remain invariant if we just interchange the values of a_1 and a_2. This implies that higher dissipation rate upstream and lower dissipation rate downstream would transmit the same amount of information as in the reverse case.

The total integrated information is given by

$$P = \int_{s_0}^{\infty} F\,(s)ds = \frac{Y_T}{a_1 a_2}\left[log\left(1 + \frac{a_1 a_2}{(a_2 + X_T)s_0}\right) - 1.5 + 2\frac{s_0}{s_0 + \frac{a_1 a_2}{a_2 + X_T}} - \frac{s_0^2}{\left(s_0 + \frac{a_1 a_2}{a_2 + X_T}\right)^2}\right] \quad (118)$$

The energy consumption rate at a particular signal strength can be quantified as

$$E = \beta_1 x_{2s} + \beta_2 y_{2s} = \alpha_1 X_T s \frac{1}{s + a_1}\left[a_1 + \frac{\alpha_2 Y_T}{\alpha_1 X_T}\frac{a_2}{\frac{s}{s + a_1} + \frac{a_2}{X_T}}\right] \quad (119)$$

The information per unit energy consumption rate

$$\frac{F}{E} = \frac{1}{\alpha_1 X_T} Y_T \frac{b^3}{a_1 a_2(a_1 + \gamma a_2)}\frac{s + a_1}{s(s+b)^3(s+c)}; b = \frac{a_1 a_2}{(a_2 + X_T)}, \gamma = \frac{\alpha_2 Y_T}{\alpha_1 X_T}, c = \frac{\gamma a_1 a_2}{(\gamma a_2 + a_1)} \quad (120)$$

$$\frac{F}{E} = \frac{1}{\alpha_2}\frac{b^3}{a_1 a_2^2}\frac{1}{s(s+b)^3}; b \approx \frac{a_1 a_2}{(X_T)}, \gamma \gg 1, c = a_1 \quad (121)$$

The expression of information per energy is not symmetric anymore because of the a_2^2 term in the denominator. This implies that if the values of a_1 and a_2 are interchanged, in the case where the downstream dissipation rate a_2 is higher the information per unit energy consumption would be lower. This corroborates our observation in the previous section that the dissipation at the lower level is more expensive with respect to energetic cost.

The integrated information per unit energy is given by

$$R = \int_{s_0}^{\infty} \frac{F(s)}{E(s)} ds = \frac{1}{\alpha_2 a_1 a_2^2}\left[log\left(1 + \frac{a_1 a_2}{X_T s_0}\right) - 1.5 + 2\frac{s_0}{s_0 + \frac{a_1 a_2}{X_T}} - \frac{s_0^2}{\left(s_0 + \frac{a_1 a_2}{X_T}\right)^2}\right] \quad (122)$$

Supplementary Figure 15B displays the patterns of information and information per energy as a function of the upstream and downstream dissipation rates ($X_T = 100; s_0 = 0.1$). It illustrates that the optimum downstream dissipation rate moves to a lower value compared to the dissipation rate upstream, when the trade-off between the information and energy is considered. The information is symmetric with respect to the dissipation rates while the information per unit energy consumption

attains an optimum where downstream dissipation rate is lower than the dissipation rate upstream. Here we did not include any noise suppression by the negative feedback in this analytical calculation of determining information transmission. Information transmission with the feedback loops is incorporated in detail in the simulation framework presented in Supplementary Note 2.

Supplementary Note 5: Estimation of energetic cost for signalling

Energy consumption for Fus3 re-phosphorylation: Since the phosphorylation reactions are essentially irreversible, ATP will be consumed at every phosphorylation event. At steady state, the ATP turnover rate would be equal to the de-phosphorylation flux. According to the reaction rate values, the ATP turnover rate is approximately 30 sec^{-1} [3] if we assume 100 molecules of the phosphatase [4]. If 50% of Fus3 is phosphorylated at steady state, and assuming that the number of Fus3 molecules is 10,000 $cell^{-1}$ [4], the ATP consumption rate is given by

$$ATPturnover = 30/s\,; phosphorylation = 5 \times 10^3;$$
$$ATP\ consumption/hr = (30/s) \times (5 \times 10^3) \times (3600\, s/hr) \sim 5 \times 10^8$$

Clearly, if the phosphatase concentration increases by two-fold, the ATP turnover rate would also increase by two-fold to keep the phosphorylation state constant.

Total energy consumed by a cell during maximum growth: Glucose uptake rate ~$10\ mmol\ gDW^{-1}h^{-1}$ and dry weight (DW) of a haploid yeast cell ~$15\, pg = 15 \times 10^{-12} g$ [6]

Number of ATP molecules produced during fermentative growth per molecule of glucose ~2 [6,7].

Thus, total ATP consumed per cell in 1 hr is given by $2 \times (10^{-2} mol g^{-1} h^{-1}) \times (6 \times 10^{23} mol^{-1}) \times (15 \times 10^{-12} g) \sim 2 \times 10^{11}$

Thus, energy consumed for Fus3 re-phosphorylation under the described assumptions approximates $\frac{5 \times 10^8}{2 \times 10^{11}} \sim 0.002$ or 0.2% of total cellular energy consumption.

Pathway protein synthesis cost: Numbers of pathway protein molecules/cell in the induced state are roughly [4]:

Fus3 ~ 10^4; Ste2 ~ 5×10^3; Ste11 ~ 3×10^3; Ste7 ~ 10^3; Ste12 ~ 10^3; Msg5 ~10^2

We assume that the proteins consist of 400 amino acids on average and approximately 5 ATP molecules are consumed per residue [6] for protein production. If we further assume that the whole set of proteins has to be newly synthesized per hour (due to growth dilution and protein degradation) then the total amount of ATP consumed per cell in one hour is given by

$$5 \times 400 \times (10^4 + 5 \times 10^3 + 3 \times 10^3 + 10^3 + 10^3 + 10^2) \sim 4 \times 10^7$$

Supplementary Figures

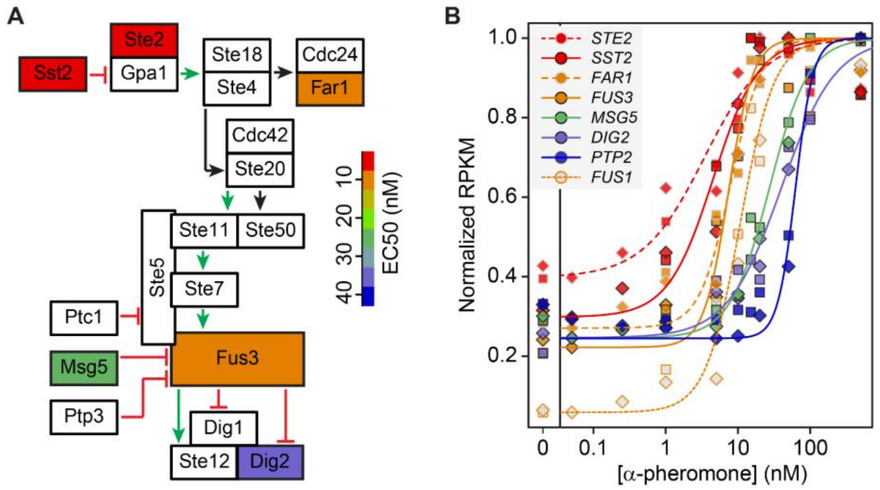

Supplementary Figure 1. Transcriptional feedback regulation of the pheromone response pathway.

(A) Schematic depiction of the yeast pheromone pathway, with pheromone-dependent transcriptional induction of pathway genes coloured according to EC50 values of their dose-responses. mRNA levels of pathway components in white boxes were not upregulated by pheromone, and these components were thus not considered as transcriptional feedback regulators.

(B) Dose-dependence of pheromone activation for indicated genes encoding pathway components. Shown are normalized RNA levels as measured in two independent RNA sequencing experiments (shown with different symbols) at 60 minutes after addition of respective dose of pheromone in a strain deleted for α-pheromone protease gene *BAR1* (Supplementary Table S1). Lines are fits with a sigmoidal function used to infer EC50 values, used for the colour scale in (A) and (B). Expression of pheromone-responsive gene *FUS1*, the promoter of which was used as a transcriptional reporter of pathway activity, is shown for comparison.

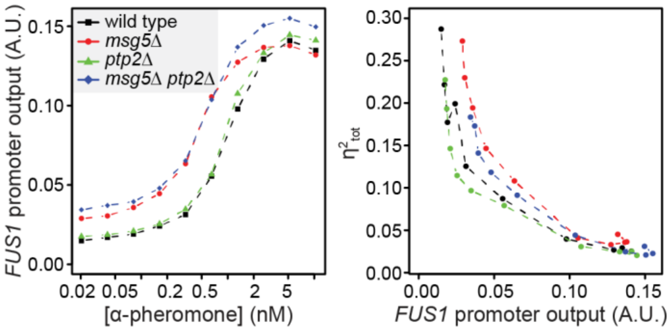

Supplementary Figure 2. Pheromone-inducible phosphatase Ptp2 has little effect on pheromone signalling.

P_{FUS1}-GFP reporter activation by different doses of pheromone (left) and noise at different reporter output levels (right) for wild type and strains deleted for phosphatase-coding genes *MSG5* and *PTP2*, separately and in combination. The experiment was performed once.

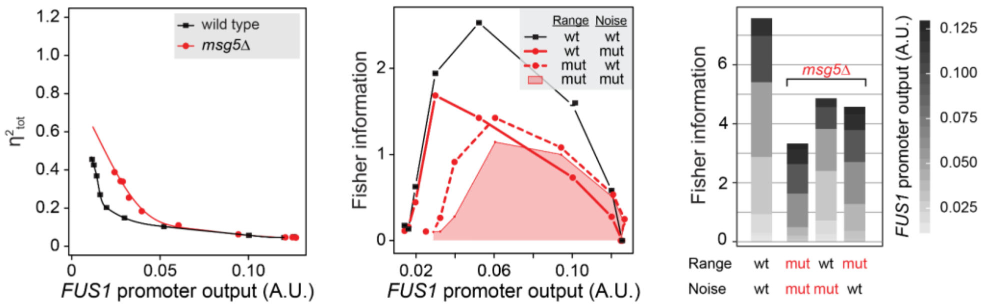

Supplementary Figure 3. Reduced output range and increased noise lower Fisher information in *msg5Δ* mutant.

Left panel: Dependence of noise on pathway output for the wild type and *msg5Δ* mutant. All analyses were performed on means of two independent experiments (shown in Fig. 2A,B). Data were first smoothed by local regression using the *loess()* function in R with standard settings and subsequently fitted with a cubic spline algorithm with 1000 points resolution using the spline() function in R, which also enabled projecting noise levels beyond the output range of *msg5Δ* mutant. Middle panel: For calculation of Fisher information with different combination of output range and noise, original data for dose responses and noise levels derived from the fits to the data were used. Thus, plots for wild type and *msg5Δ* mutant slightly differ from those shown in Fig. 2C. Projecting either wild-type output range or wild-type noise levels on the mutant increases Fisher information to a similar overall extent, but at different output ranges. Right panel: Aggregated Fisher information for the different output range / noise combinations with local information at different output levels shown in different shades of grey.

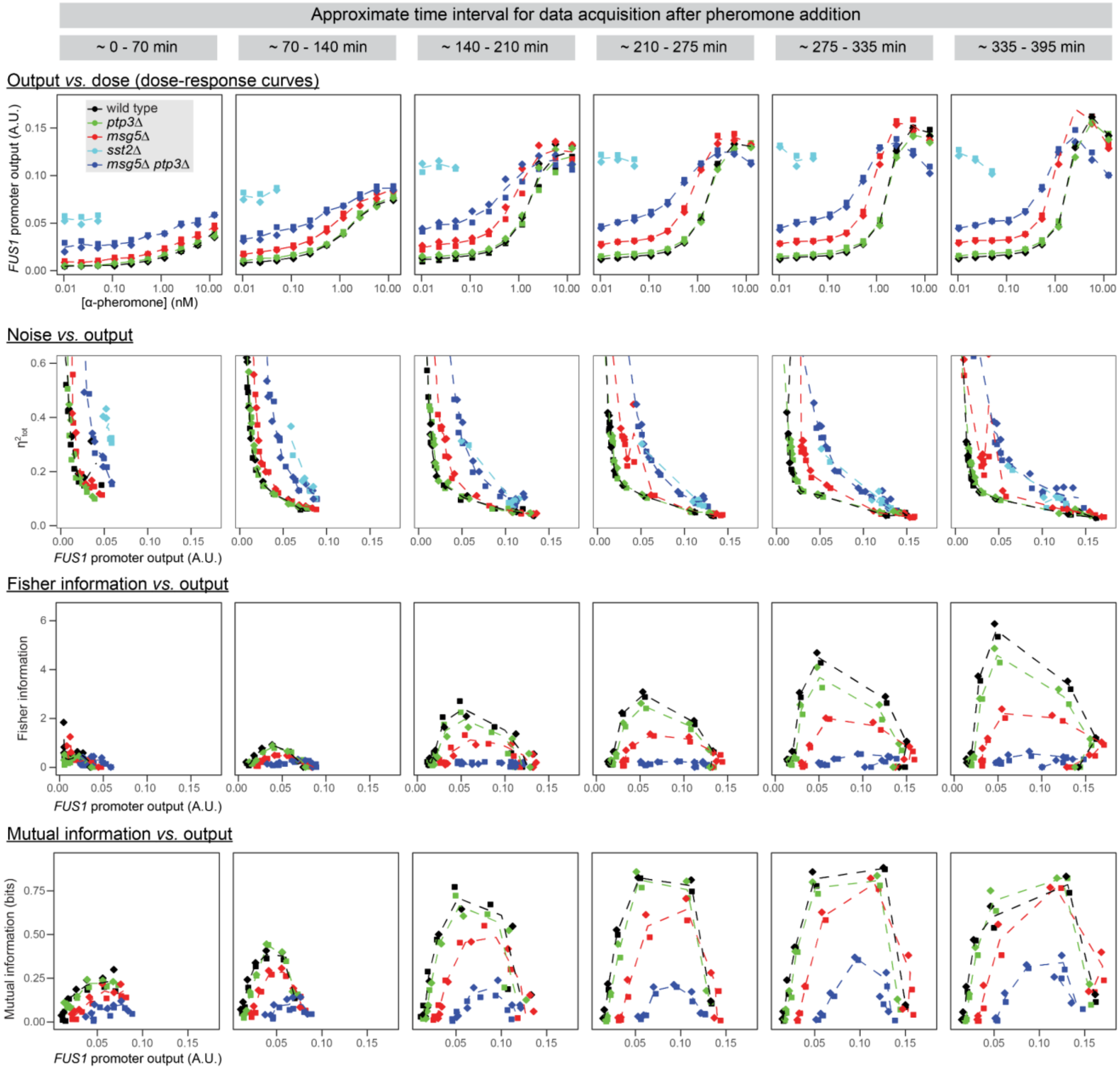

Supplementary Figure 4. Effects of negative feedback regulators over time.

Dose responses, noise, Fisher and mutual information (as indicated) at different time intervals (indicated at top) after pheromone stimulation for wild type (black) and strains deleted for negative regulators (colours as indicated). Data were obtained in two independent experiments (shown with different symbols). Dashed lines connect the means of both experiments. Plots in the third column (140-210 minutes after stimulation) are replicates of plots shown in Figure 2A-D.

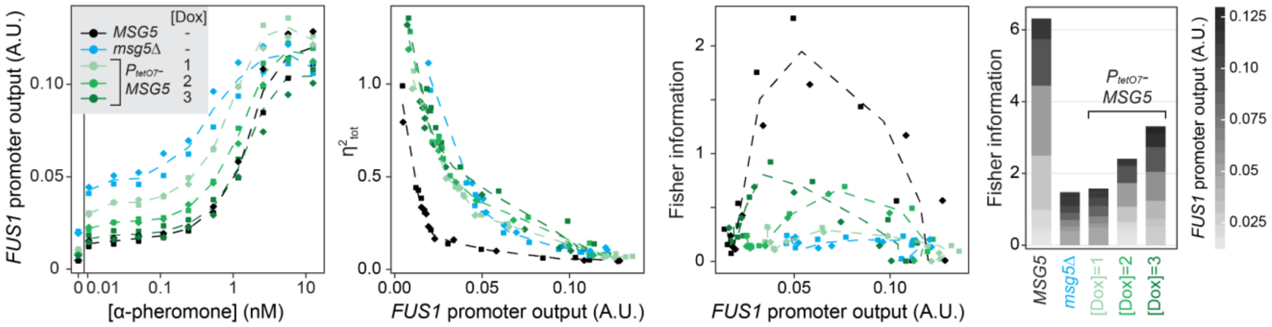

Supplementary Figure 5. Constitutive expression of *MSG5* only partially rescues loss of precision of input estimation.

Dose-dependence of pathway activation (left panel), output noise (2nd panel) and calculated Fisher information (3rd and rightmost panels) for strains with wild type (black), deletion (blue), and different levels of constitutive expression of *MSG5* (at indicated induction by doxycycline (Dox, in µg/ml)). All strains were additionally deleted for *PTP3*. The three panels on the left show results of two independent experiments (with different symbols); dashed lines connect means of both experiments. The right-most plot shows aggregated Fisher information over the whole output range as calculated from the means of both experiments, with individual contributions at different output levels in different shades of grey.

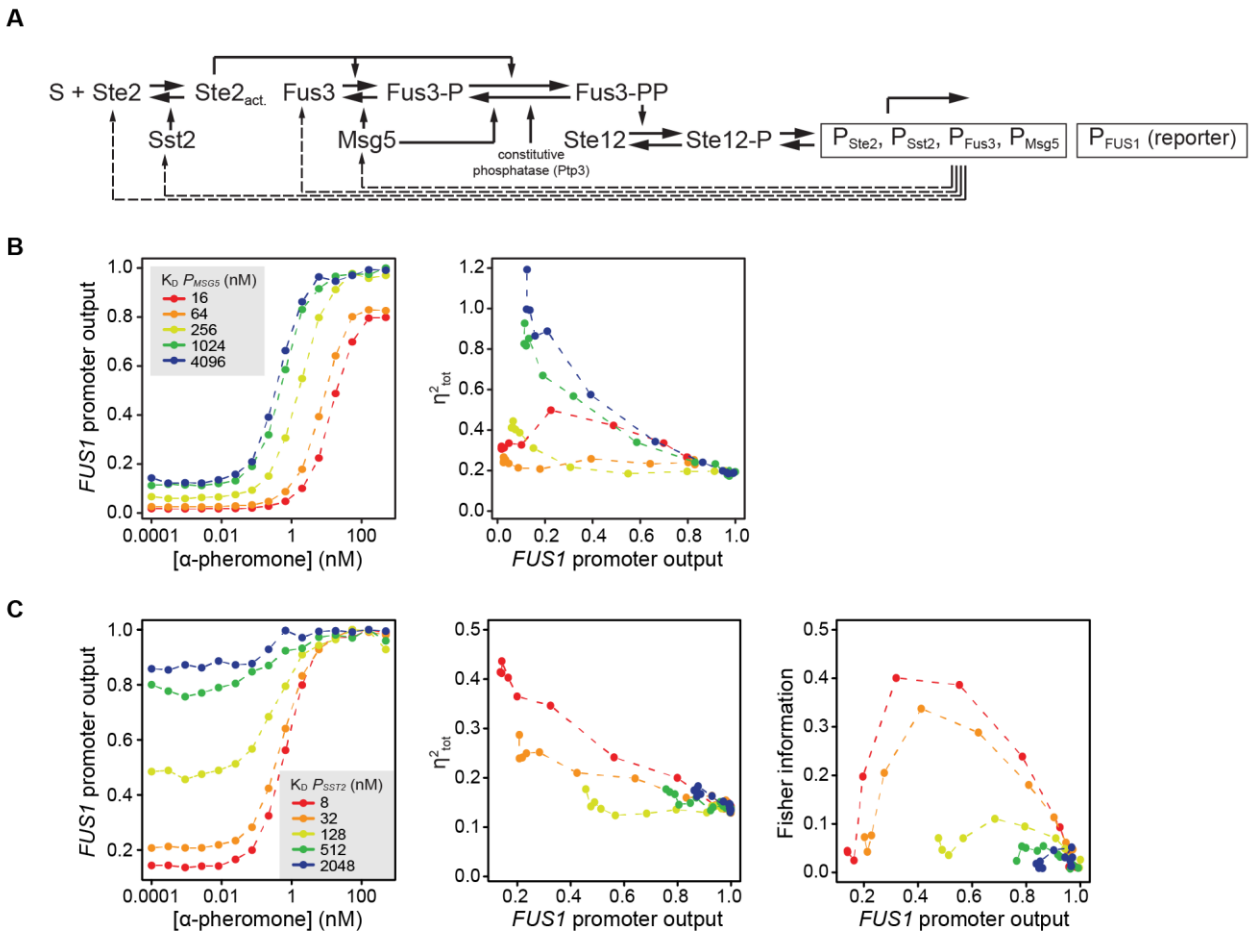

Supplementary Figure 6. Simulated effects of varying sensitivities of transcriptional *MSG5* and *SST2* induction.

(A) Cartoon of simplified mathematical model used do simulate pheromone pathway responses. Pheromone (S) binds to the receptor (Ste2) and activates Fus3 through two-step phosphorylation. Activated Fus3 further phosphorylates Ste12 which then stimulates transcription of feedback regulators Ste2, Sst2, Fus3 and Msg5 through binding of their respective promoters.

(B) Simulated pathway response (left) and noise (middle) at different sensitivities of *MSG5* induction, tuned by changing binding affinity of active Ste12-P to the *MSG5* promoter (K_D P_{MSG5}). Induction sensitivity for *SST2* was kept constant at K_D P_{SST2} = 8 nM, the value estimated from simulations of wild type data (Fig. 2). Information transmission calculated based on these data is shown in Figure 3A.

(C) Simulated pathway response (left), noise (middle) and information transmission (right) at different sensitivities of *SST2* induction. Induction sensitivity for *MSG5* was kept at the value estimated from simulating wild type data (K_D P_{MSG5} = 700 nM).

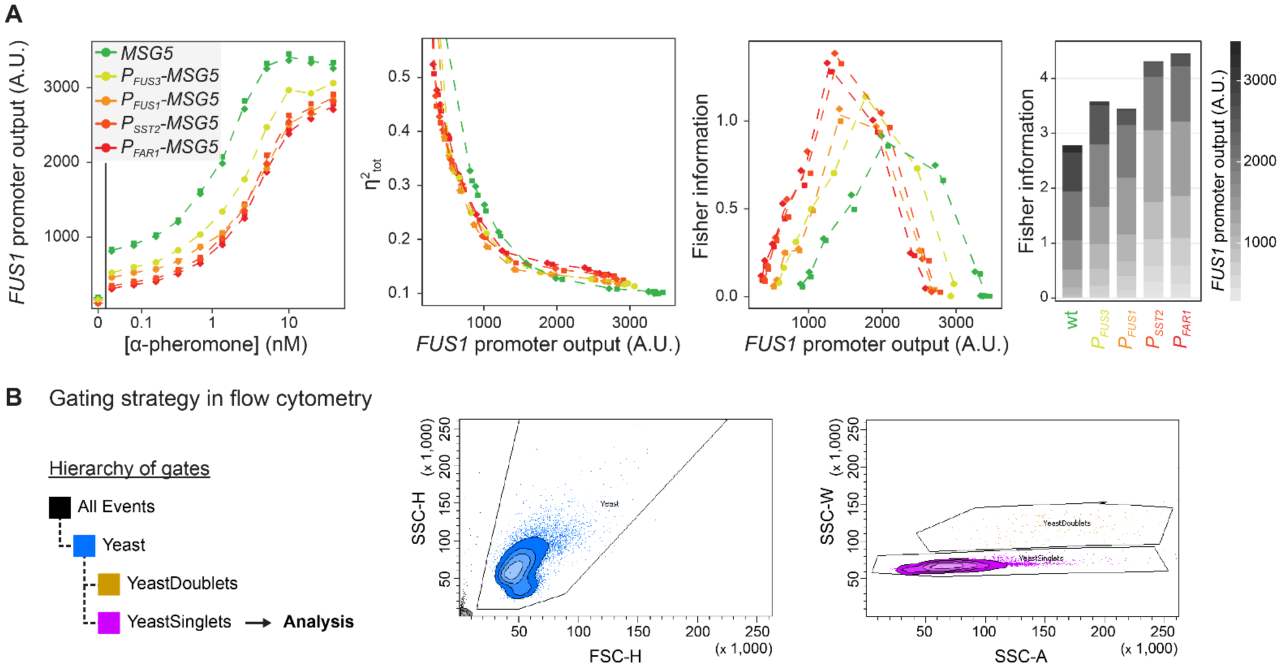

Supplementary Figure 7. Sensitized *MSG5* induction leads to higher maximal information transmission.

(A) Dose-response behaviour of P_{FUS1}-GFP (left panel), and corresponding noise (2nd panel) and Fisher information (3rd and rightmost panels) for strains in which expression of *MSG5* was controlled by its native promoter (green) or several different promoters that respond more sensitively to pheromone (yellow to red). Data were acquired by flow cytometry approximately 2.5 hours after pheromone addition with approximately 10,000 cells analysed per sample. The three plots on the left show results of two independent experiments (with different symbols); dashed lines connect means of both experiments. The right-most plot shows aggregated Fisher information over the whole output range as calculated from the means of both experiments, with individual contributions at different output levels in different shades of grey.

(B) Gating strategy for analysis of flow cytometry data, with the hierarchy of gates (left panel) and examples of plots with the defined gates (middle and right panels). The total of detected events (All Events) were gated by side scatter (SSC-H) and forward scatter (FSC-H) to define yeast cell events (Yeast gate in the middle); this population was further gated by side scatter width (SSC-W) and side scatter area (SSC-A) to distinguish yeast single cells (YeastSinglets) from yeast aggregates (YeastDoublets; plot on the right). Data shown in (A) were derived from gate YeastSinglets. Gates were drawn manually using the BD FACS DIVA software (BD Biosciences). Per sample, 10,000 events within the Yeast gate were recorded.

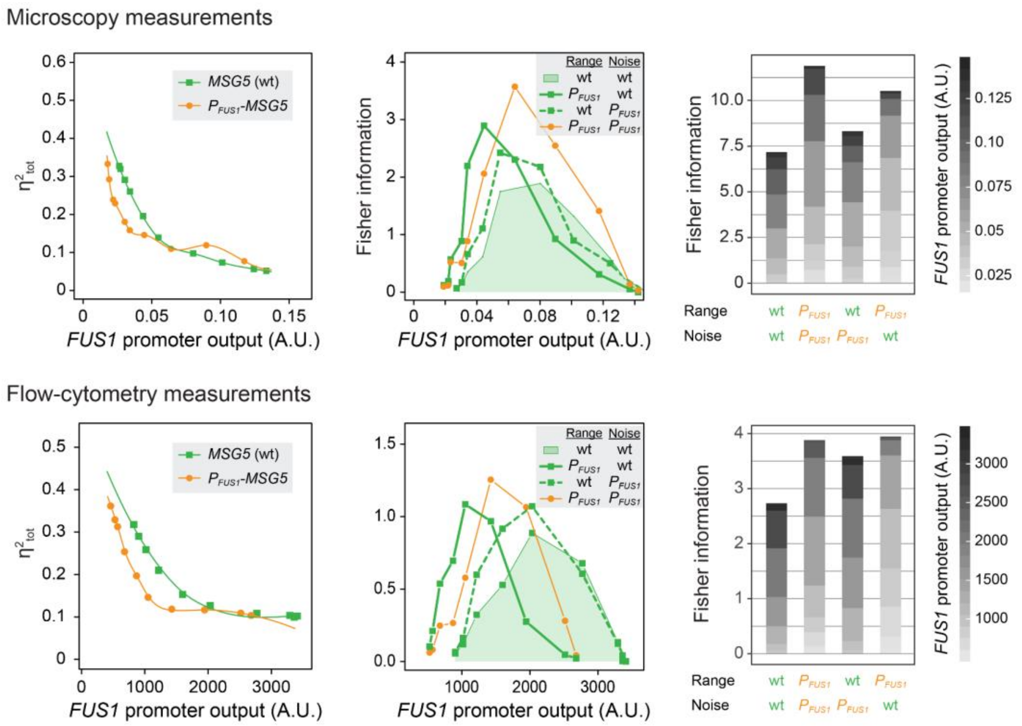

Supplementary Figure 8. Effects of output range and noise on Fisher information upon sensitized *MSG5* induction.

Approach for disentanglement and panels from left to right are as in Supplementary Figure 3. Upper and lower rows show analyses for data acquired with microscopy and flow cytometry, respectively.

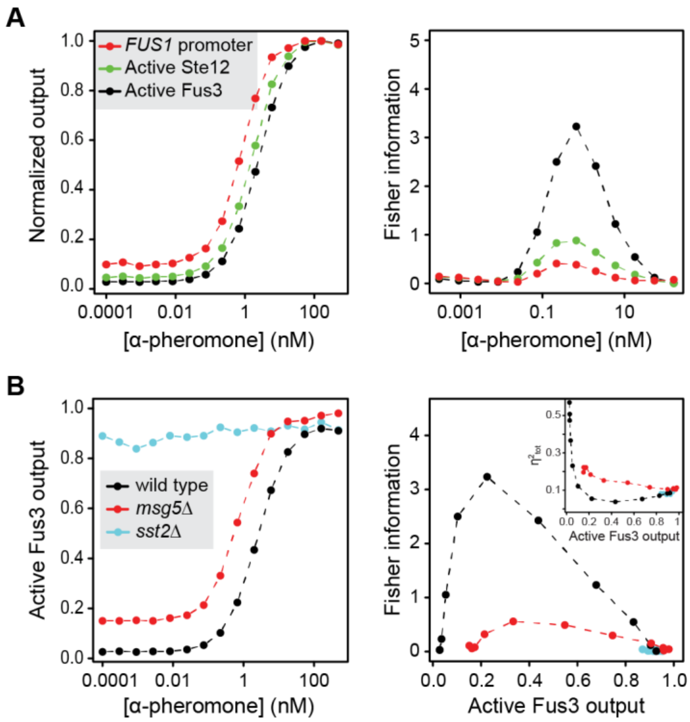

Supplementary Figure 9. Active Fus3 contains more information than a single transcriptional reporter.

(A) Simulated dose response (left panel) and Fisher information (right panel) for active Fus3 (Fus3-PP), active Ste12 (Ste12-P) and P_{FUS1} transcriptional reporter (colours as indicated).

(B) Simulation of output range (left panel) and Fisher information (right panel) and noise (right panel inset) for active Fus3 in the absence of indicated negative feedback regulators.

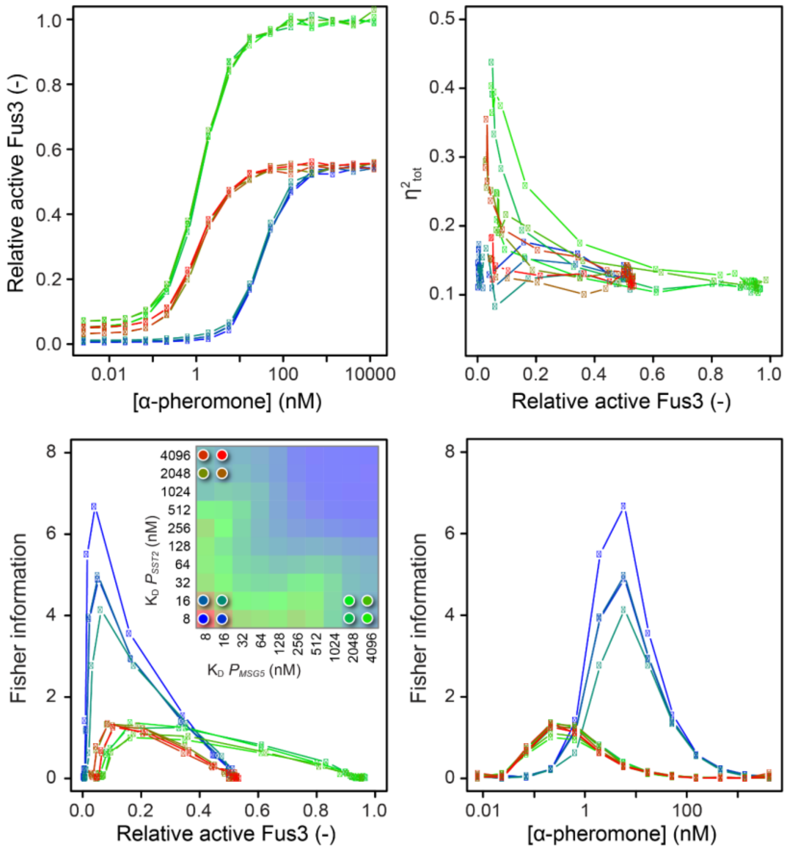

Supplementary Figure 10. Simulation of Fus3 signalling output at different *MSG5* and *SST2* induction sensitivities.

Plots show simulations of dose responses (top left panel), noise (top right panel) and Fisher information plotted against Fus3 activity (bottom left panel) or against pheromone concentrations on a logarithmic scale (bottom right panel), with selected induction sensitivities for *MSG5* and *SST2* feedbacks. The colour code for *MSG5* and *SST2* induction sensitivities, i.e., K_D values of Ste12-P to their respective promoters, is indicated in the inset of bottom left figure, which is a replicate of the Total Fisher information matrix shown in Figure 4A. Combinations of either low sensitivity for *SST2* and high sensitivity for *MSG5* (blue shades) or vice versa (green shades, corresponding to experimentally observed behaviour) result in similar total Fisher information. Concentrations of active Fus3 (Fus3-PP) were normalized to its maximum reached in all simulations.

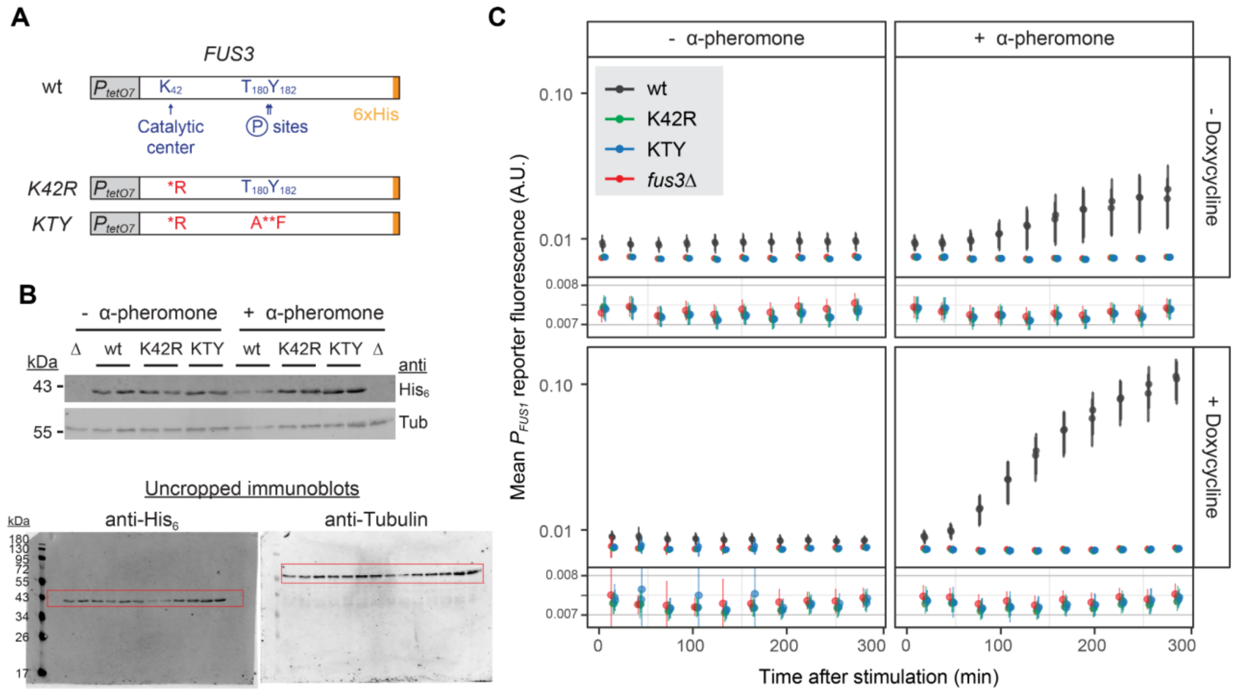

Supplementary Figure 11. Fus3 variants KTY and K42R are incapable of signalling .

(A) Schematic overview of the *FUS3* gene constructs employed for growth competition experiments. Genes are controlled by P_{tetO7} promoter, which renders them inducible by Doxycycline (Dox), and contain a C-terminal 6xHis tag. Amino acids exchanged in *fus3-K42R* and *–KTY* are indicated in red.

(B,C) Fus3-K42R and –KTY are expressed but incapable of transmitting the pheromone signal. Tested strains carried a P_{FUS1}-mCherry reporter and were either deleted for *FUS3* (Δ, yAA373) or contained a chromosomally integrated variant as described in (A). The *fus3Δ* parental strain and two transformants for each *fus3* allele were grown in minimal medium in the absence or presence (20 µg/ml) of Dox for about 5 hours. Part of the cultures was used for fluorescence time-lapse microscopy to measure expression of the pheromone-responsive P_{FUS1}-mCherry reporter in the absence or in presence (20 nM) of pheromone; activities of the reporter (mean ± s.d. of individual cells) were plotted on a logarithmic scale (C). Note that activities of the reporter in Fus3-K42R or –KTY containing strains do not change, neither by induction of *fus3*-allele expression with pheromone nor by pheromone application and are indistinguishable from each other and *fus3Δ* strain. To highlight this point, reporter activities in those strains are plotted in a zoomed-in linear scale below the main plots. The remaining Dox-containing cultures were split and incubated for another 90 min in the absence or in presence (20 nM) of pheromone. Cells were harvested and lysed and the resulting cell lysates were separated by SDS-PAGE, blotted on a Nitrocellulose membrane and probed sequentially with anti-His (Biolegend) and anti-Tubulin (Abcam) antibodies; uncropped full scans of the immunoblot membrane are shown at the bottom (B). Experiments shown in (B,C) were performed once.

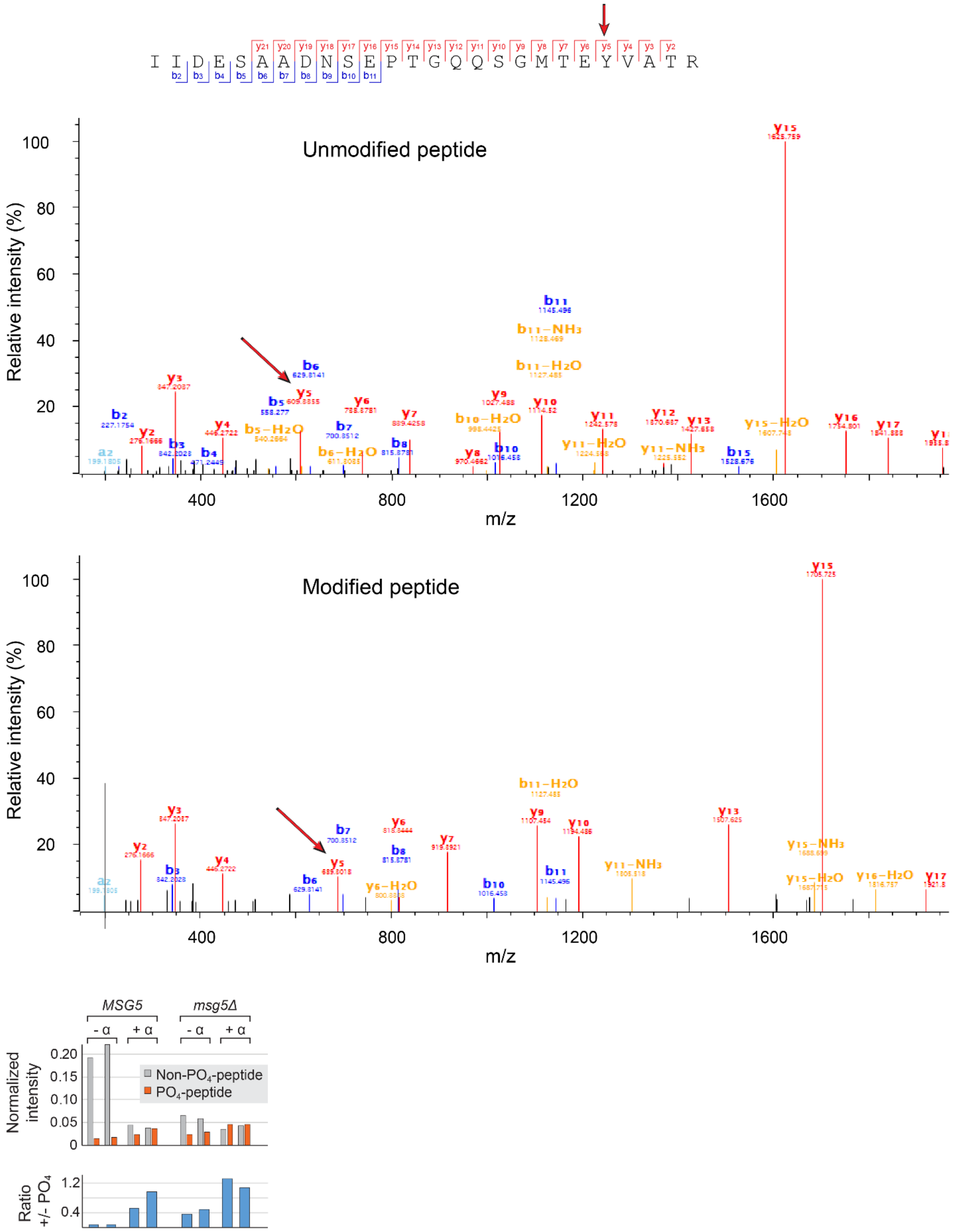

Supplementary Figure 12 . *In-vivo* phosphorylation of Fus3-K42R.

Strains expressing Fus3-K42R (two biologically independent strains for each wild type *MSG5* and *msg5Δ*) were grown in the in absence (-α) or presence of 20 nM (+α) pheromone for 3 hours. Proteins were extracted and enriched for His_6-tagged Fus3 protein under denaturing conditions using a quick extraction protocol[8] and pre-packed Nickel columns (Protino Ni-TED from MACHEREY-NAGEL). Samples were analysed by Liquid Chromatography-Mass Spectrometry followed by data analysis as

described previously[9]. Sequence of the Fus3 peptide containing the phosphorylation sites is shown at the top; the two plots below show MS/MS spectra obtained for this peptide, either unmodified or phosphorylated. Identified b- and y-fragment ions are shown in blue and red, respectively, in the sequence as well as in the spectra. The y5 ion (marked with red arrows in the sequence and spectra) of the y-ion series allowed identification of the phosphorylation modification on Tyrosine Y_{182}. The mass difference of the y5 ion of the modified versus of the unmodified peptide is 80 Da, corresponding to the mass of a phosphate group. Label-free quantification of the samples was performed using MaxQuant (Version 1.6.7.0, details for settings can be provided upon request)[10]. Plots at the bottom display quantifications of Phosphate-group containing (orange) and non-containing (grey) peptides normalized to the amount of total Fus3 protein and the corresponding ratios between modified and unmodified peptides (blue columns).

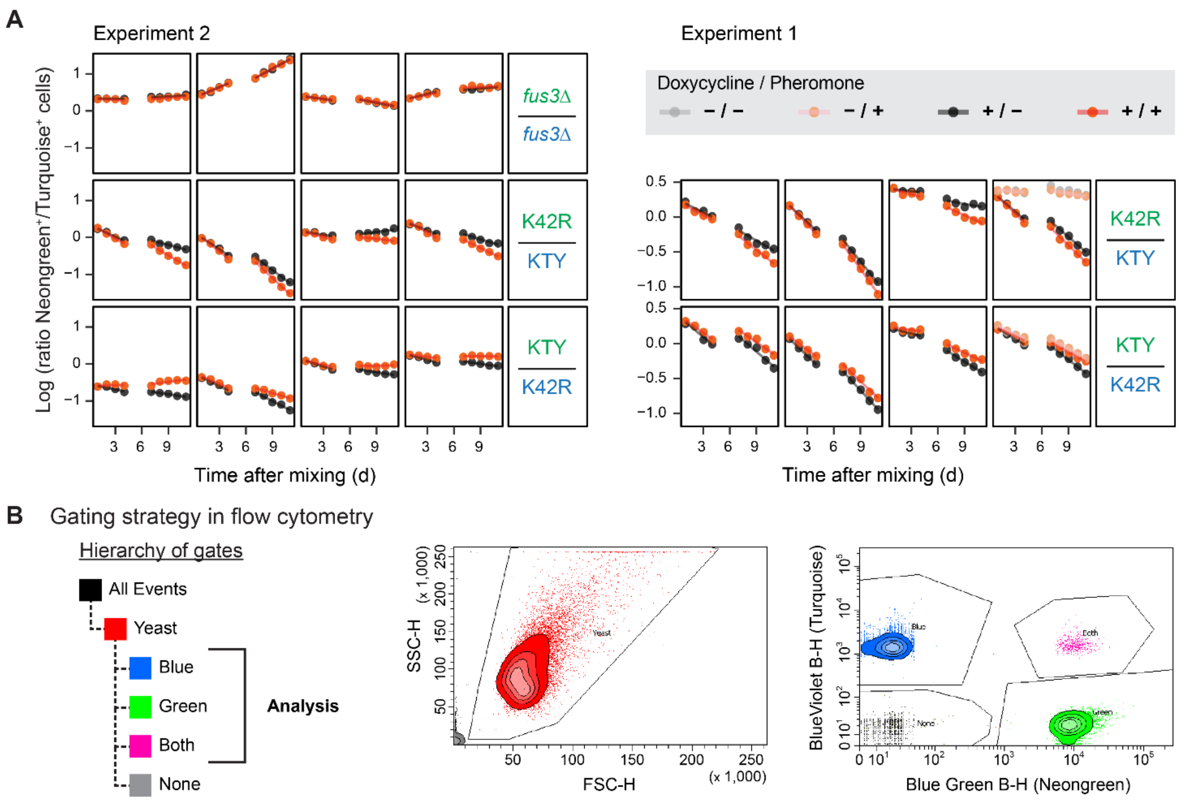

Supplementary Figure 13. Growth in presence of pheromone favours Fus3-KTY versus -K42R expressing cells when compared to the growth in absence of pheromone.

(A) Shown are ratios ($R_{Ng/Tq}$) between mNeongreen (Ng) and mTurquoise (Tq) expressing cells in medium without (black) or with pheromone (red, 20 nM) over time for two independent experiments (see labels above plots). Every co-culture contained an Ng and a Tq expressing strain. Strains in different co-cultures were independent transformants isolated after transformation with *fus3* allele containing (for *fus3-K42R* and *-KTY*) or fluorescence reporter containing (for *fus3Δ*) integrative plasmids. *fus3* variants co-expressed with Ng (above fraction bar) and Tq (below fraction bar), respectively, are shown on the right side of the panels. Co-cultures were continuously grown over a period of about two weeks with 2.5 days interruption when cultures were stored at 4°C. Re-inoculation into fresh media was carried out twice a day and flow-cytometry measurements to determine ratios of Ng to Tq expressing cells once a day. Summaries of the results are shown in Figure 5B, where the interruption-period was omitted to account exclusively for periods in which cells were growing. Experiment 2 was carried out with control mixtures containing strains deleted for *FUS3*, experiment 1 was carried out with control mixtures in which *fus3* variants were not induced (light black and light red traces) by Dox. Note that for control mixtures traces in the absence and presence of pheromone do not diverge from each other, whereas for mixtures with cells expressing the two different *fus3* variants they do.

(B) Gating strategy for analysis of flow cytometry data with the hierarchy of gates (left) and examples of plots with the defined gates (middle and right). The total of detected events (All Events) were gated by side scatter (SSC-H) and forward scatter (FSC-H) to define yeast cell events (Yeast gate in the middle); this population was further gated by mTurquoise (BlueViolet B-H) and mNeongreen (Blue Green B-H) fluorescence to define populations expressing either one (gates Blue and Green, respectively) or both fluorescent proteins (gate Both, plot on the right). The ratio of Neongreen- and Turquoise-expressing cells was calculated as ($\#_{Green}$ + $\#_{Both}$) / ($\#_{Blue}$ + $\#_{Both}$) with $\#_{Gate}$ denoting the

number of cells within the gate. Gates were drawn manually with the BD FACS DIVA software (BD Biosciences). Per sample, 20,000 events within the Yeast gate were recorded.

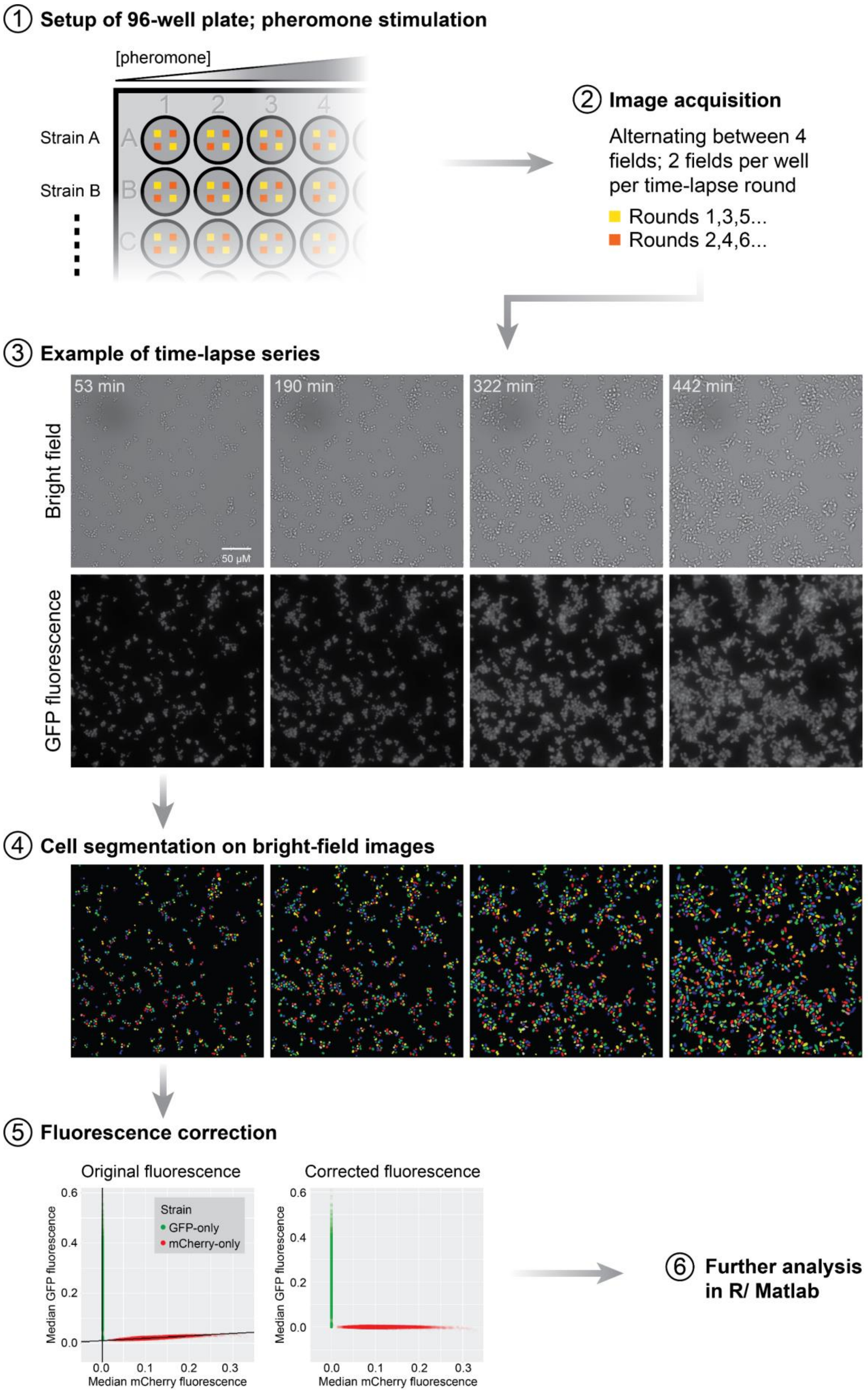

① Setup of 96-well plate; pheromone stimulation
[pheromone]
1
2
3
4
Strain A
Strain B
A
B
C
② Image acquisition
Alternating between 4 fields; 2 fields per well per time-lapse round
Rounds 1,3,5...
Rounds 2,4,6...
③ Example of time-lapse series
Bright field
GFP fluorescence
53 min
190 min
322 min
442 min
50 μM
④ Cell segmentation on bright-field images
⑤ Fluorescence correction
Original fluorescence
Corrected fluorescence
Strain
GFP-only
mCherry-only
Median GFP fluorescence
Median mCherry fluorescence
0.0
0.2
0.4
0.6
0.1
0.3
⑥ Further analysis in R/ Matlab

Supplementary Figure 14. Experimental and data processing workflow for single-cell analysis.

(1) Typical setup for microscopy in a 96-well glass-bottom plate. Different strains, typically expressing super-folder GFP (P_{FUS1} reporter) and mCherry (constitutive reporter), were pre-grown in parallel, transferred to the plate and stimulated with different concentrations of pheromone. Routinely, each plate contained control strains expressing one fluorescent protein (not shown).

(2) Microscopy was started immediately after pheromone application. Two fields were imaged per well in one time-lapse round, with alternating pairs in even and uneven rounds (see differently coloured squares), resulting in a total of four fields per well. Imaging of a whole plate took approximately one hour; the order of acquisition was chosen such that wells with same pheromone concentrations were imaged successively. Typically, acquisition proceeded for 8-12 rounds.

(3) Example of a time-lapse series of bright-field and GFP fluorescence images of one field of view. Scale bar corresponds to 50 µM.

(4) Example of cell segmentation (i.e., detection of single cells) as done on the bright-field images shown above using CellProfiler. The defined masks were used for measuring fluorescence intensities in the corresponding fluorescence images; median intensities for each detected cell were exported from CellProfiler for further processing. Scale is the same as in (3).

(5) Routinely, processing of raw fluorescence data entailed correction for autofluorescence and bleed-through between different fluorescence channels based on control strains expressing only one fluorescent protein. Furthermore, upper and lower 3-percentiles per field of view and time point in either fluorescence channel were routinely removed from further analysis.

(6) Corrected values were used for further analysis with R and/or Matlab.

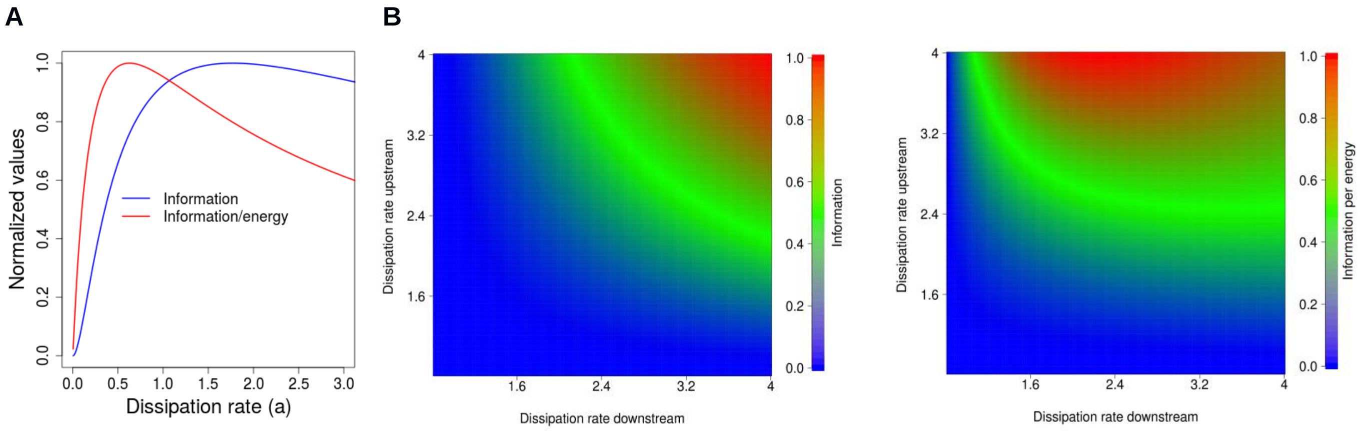

Supplementary Figure 15. Patterns of information and information per energy in simple one- and two-stage phosphorylation cascades.

(A) Information and information per unit energy consumption rate in a one-stage cascade as a function of the dissipation rate *a*. Values on the Y-axis are normalized values of information (blue) and information per unit energy (red), respectively. The maximum in information per energy demonstrates a trade-off between information and energy expenditure. See Supplementary Note 4 for details.

(B) Information (left panel) and information per unit energy consumption rate (right panel) in a two-stage cascade as a function of dissipation rates at upper (Y-axis) and lower (X-axis) stages. The colour code represents normalized values of information and information per energy as indicated. The symmetry in information with respect to the upstream and downstream dissipation rates is lost for information per energy. See Supplementary Note 4 for details.

Supplementary Table 1. Yeast strains used in this study.

Strain name	Relevant genotype	Description
yAA95	*ura3::*pAA35*(P_{FUS1}-Ubi(I)-sfGFP-T_{FUS1} :URA3) bar1Δ::kanMX6 mfα1Δ::SpHIS3 mfα2Δ::hphNT1 arg4Δ::klTRP1*	P_{FUS1} destabilized superfolder-GFP reporter integrated in *ura3* locus via integrative plasmid (pAA35); *bar1*, *mfα*1/2 and *arg4* deletions; used for RNA sequencing experiments
yAA248	*mfα2Δ::[P_{tetO7}-Ubi(I)-sfGFP-T_{AGA2}:URA3] LYS2:P_{ADH1}-rtTAS2-T_{ADH1} mfα1Δ::klTRP1 bar1Δ::natNT2*	Reverse tetra/doxycyline controlled transactivator (rtTAS2); tetO7 driven sfGFP; used as GFP-only control for microscopy
yMFM039	*trp1 Δ::[P_{TetO7}-mCherry-T_{ADH1}:CgTRP1] LYS2:P_{ADH1}-rtTAS2-T_{ADH1}*	Reverse tetra/doxycyline controlled transactivator (rtTAS2); tetO7 driven mCherry; used as mCherry-only control for microscopy
yAA288	*ura3::pAA35(P_{FUS1}-Ubi(I)-sfGFP-T_{FUS1} :URA3) mfα2Δ::[P_{TDH3}-mCherry-T_{TDH3} :HIS3] mfα1Δ::klTRP1 bar1Δ::natNT2 LYS2:P_{ADH1}-rtTAS2-T_{ADH1}*	P_{FUS1} destabilized superfolder-GFP reporter; constitutive (*TDH3* promoter controlled) mCherry reporter; *bar1* and *mfα*1/2 deletions; reverse tetra/doxycyline controlled transactivator (rtTAS2); served as **"wild type" for microscopy** and flow cytometry **experiments**
yAA373	*lys2ΔC:[pADH1-rtTAS2-ADH1t:LYS2C] fus3Δ::hphNT1 bar1Δ::[P_{FUS1}-mCherry-T_{FUS1}:HIS3]*	*bar1* and *fus3* deletions; P_{FUS1} mCherry reporter; reverse tetra/doxycyline controlled transactivator (rtTAS2); served as **basis for** strains constructed for **growth competition experiments**
The following strains are derivatives of yAA288. Genotypes and descriptions are given relative to yAA288		
yAA290	*msg5Δ::kanMX*	*msg5* deletion
yAA305	*ptp3Δ::LEU2*	*ptp3* deletion
yAA306	*ptp3Δ::LEU2 [kanMX:P_{tetO7}]::MSG5*	*ptp3* deletion; insertion of P_{tetO7} promoter upstream of chromosomal *MSG5* to control *MSG5* expression by doxycycline
yAA307	*msg5Δ::kanMX ptp3Δ::LEU2*	*msg5* and *ptp3* deletions
yAA318	*sst2Δ::LEU2*	*sst2* deletion; displays growth defect, likely due to strongly elevated basal pheromone pathway activity
yAA410	*[kanMX:P_{FAR1}]::MSG5*	*FAR1* promoter upstream of *MSG5* coding sequence
yAA411	*[kanMX:P_{FUS1}]::MSG5*	*FUS1* promoter upstream of *MSG5* coding sequence
yAA412	*[kanMX:P_{FUS3}]::MSG5*	*FUS3* promoter upstream of *MSG5* coding sequence
yAA414	*[kanMX:P_{SST2}]::MSG5*	*SST2* promoter upstream of *MSG5* coding sequence
The following strains were used for growth-competition experiments and are derivatives of yAA373. Genotypes and descriptions are given relative to yAA373.		
yAA417-1 to yAA417-4	*leu2Δ::[P_{tetO7}-fus3-K42R-His_6:CgLEU2] ura3::pAA232(P_{TDH3}-mNeongreen-T_{TDH3}:URA3)*	Chromosomally integrated P_{tet} controlled and C-terminally His_6-tagged *fus3-K42R* allele; constitutively expressed mNeongreen (different transformants picked after *fus3-K42R* and prior to mNeongreen integration)

yAA417-6 to yAA417-9	*leu2Δ::[P_{tetO7}-fus3-K42R-His$_6$:CgLEU2] ura3::pMFM025(P_{TDH3}-yomTurquoise- T_{TDH3}:URA3)*	Chromosomally integrated P_{tet} controlled and C-terminally His$_6$-tagged *fus3-K42R* allele; constitutively expressed mTurquois (different transformants picked after *fus3-K42R* and prior to mTurquois integration)
yAA418-1 to yAA418-4	*leu2Δ::[P_{tetO7}-fus3-KTY-His$_6$:CgLEU2] ura3::pMFM025(P_{TDH3}-yomTurquoise- T_{TDH3}:URA3)*	Chromosomally integrated P_{tet} controlled and C-terminally His$_6$-tagged *fus3-KTY* allele; constitutively expressed mTurquois (different transformants picked after *fus3-KTY* and prior to mTurquois integration)
yAA418-6 to yAA418-9	*leu2Δ::[P_{tetO7}-fus3-KTY-His$_6$:CgLEU2] ura3::pMFM025(P_{TDH3}-mNeongreen-T_{TDH3}:URA3)*	Chromosomally integrated P_{tet} controlled and C-terminally His$_6$-tagged *fus3-KTY* allele; constitutively expressed mNeongreen (different transformants picked after *fus3-KTY* and prior to mNeongreen integration)
yAA429-1 to -4	like yAA417-1 to -4, with *msg5Δ::kanMX*	Derivatives of strains used for growth competition experiments with additional deletion of *MSG5* and *STE7*, respectively
yAA429-6 to -9	like yAA417-6 to -9, with *msg5Δ::kanMX*	
yAA430-1 to -4	like yAA418-1 to -4, with *msg5Δ::kanMX*	
yAA430-6 to -9	like yAA418-6 to -9, with *msg5Δ::kanMX*	
yAA431-1A to -4B	like yAA417-1 to -4, with *ste7Δ::kanMX*	
yAA431-6A to -9B	like yAA417-6 to -9, with *ste7Δ::kanMX*	
yAA432-1A to -4B	like yAA418-1 to -4, with *ste7Δ::kanMX*	
yAA432-6A to -9B	like yAA418-6 to -9, with *ste7Δ::kanMX*	

Supplementary References

1. Cheong, R., Rhee, A., Wang, C. J., Nemenman, I. & Levchenko, A. Information transduction capacity of noisy biochemical signaling networks. *Science* **334**, 354–8 (2011).
2. Vanlier, J., Tiemann, C. A., Hilbers, P. A. J. & van Riel, N. A. W. An integrated strategy for prediction uncertainty analysis. *Bioinformatics* **28**, 1130–5 (2012).
3. Hilioti, Z. *et al.* Oscillatory phosphorylation of yeast Fus3 MAP kinase controls periodic gene expression and morphogenesis. *Curr. Biol.* **18**, 1700–6 (2008).
4. Thomson, T. M. *et al.* Scaffold number in yeast signaling system sets tradeoff between system output and dynamic range. *Proc. Natl. Acad. Sci. U. S. A.* **108**, 20265–70 (2011).
5. Ghaemmaghami, S. *et al.* Global analysis of protein expression in yeast. *Nature* **425**, 737–41 (2003).
6. Milo, R., Jorgensen, P., Moran, U., Weber, G. & Springer, M. BioNumbers--the database of key numbers in molecular and cell biology. *Nucleic Acids Res.* **38**, D750-3 (2010).
7. Futcher, B. Metabolic cycle, cell cycle, and the finishing kick to Start. *Genome Biol.* **7**, 107 (2006).
8. von der Haar, T. Optimized protein extraction for quantitative proteomics of yeasts. *PLoS One* **2**, e1078 (2007).
9. Gómez-Santos, N., Glatter, T., Koebnik, R., Świątek-Połatyńska, M. A. & Søgaard-Andersen, L. A TonB-dependent transporter is required for secretion of protease PopC across the bacterial outer membrane. *Nat. Commun.* **10**, 1360 (2019).
10. Cox, J. & Mann, M. MaxQuant enables high peptide identification rates, individualized p.p.b.-range mass accuracies and proteome-wide protein quantification. *Nat. Biotechnol.* **26**, 1367–72 (2008).
