# Supplementary material for: Design of a MAPK signalling cascade balances energetic cost versus accuracy of information transmission
Source: Nat Commun. 2020 Jul 13;11:3494. doi: 10.1038/s41467-020-17276-4 (PMC7359329; doi:10.1038/s41467-020-17276-4)
Supplement: Supplementary file 3 — Reporting Summary [file 41467_2020_17276_MOESM3_ESM.pdf]

nature research

Corresponding author(s): Victor Sourjik
Bhaswar Ghosh
(NCOMMS-19-08753A)

Reporting Summary

Nature Research wishes to improve the reproducibility of the work that we publish. This form provides structure for consistency and transparency in reporting. For further information on Nature Research policies, see Authors & Referees and the Editorial Policy Checklist.

Statistical parameters

When statistical analyses are reported, confirm that the following items are present in the relevant location (e.g. figure legend, table legend, main text, or Methods section).

n/a	Confirmed	
☐	☒	The exact sample size (*n*) for each experimental group/condition, given as a discrete number and unit of measurement
☐	☒	An indication of whether measurements were taken from distinct samples or whether the same sample was measured repeatedly
☐	☒	The statistical test(s) used AND whether they are one- or two-sided<br>*Only common tests should be described solely by name; describe more complex techniques in the Methods section.*
☒	☐	A description of all covariates tested
☐	☒	A description of any assumptions or corrections, such as tests of normality and adjustment for multiple comparisons
☐	☒	A full description of the statistics including central tendency (e.g. means) or other basic estimates (e.g. regression coefficient) AND variation (e.g. standard deviation) or associated estimates of uncertainty (e.g. confidence intervals)
☐	☒	For null hypothesis testing, the test statistic (e.g. *F*, *t*, *r*) with confidence intervals, effect sizes, degrees of freedom and *P* value noted<br>*Give P values as exact values whenever suitable.*
☐	☒	For Bayesian analysis, information on the choice of priors and Markov chain Monte Carlo settings
☒	☐	For hierarchical and complex designs, identification of the appropriate level for tests and full reporting of outcomes
☒	☐	Estimates of effect sizes (e.g. Cohen's *d*, Pearson's *r*), indicating how they were calculated
☐	☒	Clearly defined error bars<br>*State explicitly what error bars represent (e.g. SD, SE, CI)*

Our web collection on statistics for biologists may be useful.

Software and code

Policy information about availability of computer code

Data collection	NIS Elements 4.5 (Nikon), FACSDiva (BD) 8.0.1
Data analysis	CellProfiler 2.1.1, MATLAB 2013b, R version 3 (each with custom scripts)

For manuscripts utilizing custom algorithms or software that are central to the research but not yet described in published literature, software must be made available to editors/reviewers upon request. We strongly encourage code deposition in a community repository (e.g. GitHub). See the Nature Research guidelines for submitting code & software for further information.

Data

Policy information about availability of data

All manuscripts must include a data availability statement. This statement should provide the following information, where applicable:

- Accession codes, unique identifiers, or web links for publicly available datasets
- A list of figures that have associated raw data
- A description of any restrictions on data availability

Manuscript contains "Data availability" section.

Field-specific reporting

Please select the best fit for your research. If you are not sure, read the appropriate sections before making your selection.

☒ Life sciences ☐ Behavioural & social sciences ☐ Ecological, evolutionary & environmental sciences

For a reference copy of the document with all sections, see nature.com/authors/policies/ReportingSummary-flat.pdf

Life sciences study design

All studies must disclose on these points even when the disclosure is negative.

Sample size	Microscopy: number of analyzed cells per sample and time point: 300-4,000, limited by cell density and the size of field of view. In order to asses if (minimal) sample size of 300 allows to correctly estimate gene expression (GFP) distribution of the population, we have performed tests on samples with larger cell numbers by repeatedly sampling subsets of n=300 for all used pheromone concentrations. We found that coefficients of variation for means and s.d.'s of these different subsets were smaller than 5% and 7%, respectively. For flow cytometry, cell numbers were in large excess over those numbers (10,000 cells per sample in promoter exchange experiments (Suppl. Fig. 7) and 20,000 cells per sample in growth competition experiments (Suppl. Fig. 13))
Data exclusions	Microscopy: Exclusion of upper and lower 3-percentile in each fluorescence channel in order to exclude segmentation artifacts.
Replication	At least two biological replicates. All attempts in replication were successful to reproduce major conclusions.
Randomization	n/a; data analysis had been performed automatically, thus, no human bias was possible
Blinding	n/a; data analysis had been performed automatically, thus, no human bias was possible

Reporting for specific materials, systems and methods

Materials & experimental systems

n/a	Involved in the study	
☒	☐	Unique biological materials
☒	☐	Antibodies
☒	☐	Eukaryotic cell lines
☒	☐	Palaeontology
☒	☐	Animals and other organisms
☒	☐	Human research participants

Methods

n/a	Involved in the study	
☒	☐	ChIP-seq
☐	☒	Flow cytometry
☒	☐	MRI-based neuroimaging

Flow Cytometry

Plots

Confirm that:

☒ The axis labels state the marker and fluorochrome used (e.g. CD4-FITC).

☒ The axis scales are clearly visible. Include numbers along axes only for bottom left plot of group (a 'group' is an analysis of identical markers).

☐ All plots are contour plots with outliers or pseudocolor plots.

☒ A numerical value for number of cells or percentage (with statistics) is provided.

Methodology

Sample preparation	Yeast cells growing in Synthetic minimal medium, 1:6 diluted in same medium in 96-well plate
Instrument	LSR Fortessa Special Order flow cytometer (BD Biosciences)
Software	FACSDiva (BD) for data collection and analysis, R (package flowCore) and Matlab for analysis
Cell population abundance	Yeast cell abundance in media > 1000 cells/µl. Yeast gate contained more than 80% of total event number.

Gating strategy

FSC/SSC for gating yeast cells; for growth-competition experiments gating in Neongreen/Turquoise plot to distinguish Neongreen from Turquoise expressing cells (Suppl. Fig. 13); for promoter exchange experiments gating in SSC-W/SSC-A to distinguish single cells from aggregates/doublets (Suppl. Fig. 7)

☒ Tick this box to confirm that a figure exemplifying the gating strategy is provided in the Supplementary Information.
